# LncRNA SNHG4 promotes prostate cancer cell survival and resistance to enzalutamide through a let-7a/RREB1 positive feedback loop and a ceRNA network

**DOI:** 10.1186/s13046-023-02774-2

**Published:** 2023-08-18

**Authors:** Qingzhuo Dong, Hui Qiu, Chiyuan Piao, Zhengxiu Li, Xiaolu Cui

**Affiliations:** 1 Department of Urology, First Hospital of China Medical University, #155 Nanjing North Road, Shenyang, 110001 China; 2 Department of Gynecology and Obstetrics, Shengjing Hospital of China Medical University, Shenyang, 110004 China; 3 Department of Dermatology, First Hospital of China Medical University, Shenyang, 110001 China

**Keywords:** Prostate cancer, SNHG4, RRM2, Enzalutamide resistance, ceRNA

## Abstract

**Background:**

Prostate cancer threatens the health of men over sixty years old, and its incidence ranks first among all urinary tumors among men. Enzalutamide remains the first-line drug for castration-resistant prostate cancer, however, tumors inevitably become resistant to enzalutamide. Hence, it is of great importance to investigate the mechanisms that induce enzalutamide resistance in prostate cancer cells.

**Methods:**

Bioinformatic analyzing approaches were used to identified the over-expressed genes in prostate cancer tumor tissues from three GEO datasets. qRT-PCR, western blotting and immunochemistry/In situ hybridization staining assays were performed to assess the expression of SNHG4, RRM2, TK1, AURKA, EZH2 and RREB1. Cell cycle was measured by flow cytometry. CCK-8, plate colony formation and EdU assays were performed to assess the cell proliferation. Senescence-associated β-Gal assay was used to detect the cell senescence level. γ-H2AX staining assay was performed to assess the DNA damages of PCa cells. Luciferase reporter assay and RNA immunoprecipitation assay were performed to verify the RNA-RNA interactions. Chromatin immunoprecipitation assay was performed to assess the bindings between protein and genomic DNA.

**Results:**

We found that RRM2 and NUSAP1 are highly expressed in PCa tumors and significantly correlated with poor clinical outcomes in PCa patients. Bioinformatic analysis as well as experimental validation suggested that SNHG4 regulates RRM2 expression via a let-7 miRNA-mediated ceRNA network. In addition, SNHG4 or RRM2 knockdown significantly induced cell cycle arrest and cell senescence, and inhibited DNA damage repair and cell proliferation, and the effects can be partially reversed by let-7a knockdown or RRM2 reoverexpression. In vitro and in vivo experiments showed that SNHG4 overexpression markedly enhanced cell resistance to enzalutamide. RREB1 was demonstrated to transcriptionally regulate SNHG4, and RREB1 was also validated to be a target of let-7a and thereby regulated by the SNHG4/let-7a feedback loop.

**Conclusion:**

Our study uncovered a novel molecular mechanism of lncRNA SNHG4 in driving prostate cancer progression and enzalutamide resistance, revealing the critical roles and therapeutic potential of RREB1, SNHG4, RRM2 and let-7 miRNAs in anticancer therapy.

**Supplementary Information:**

The online version contains supplementary material available at 10.1186/s13046-023-02774-2.

## Background

The incidence of prostate cancer (PCa) ranks 1^st^ among all urinary cancers in men, and PCa has become a worldwide health burden [1, 2]. In recent decades, the mechanisms driving the initiation and progression of PCa have been well characterized, and androgen receptor (AR) has been identified as a major player in PCa [3]. Therefore, therapies based on androgen deprivation have become the first-line strategy for initially diagnosed PCa patients [4, 5]. With the help of androgen-deprivation therapy (ADT), the overall survival for PCa patients in the first five years after initial diagnosis could reach 90% or even higher [4, 5]. However, most PCa patients inevitably develop castration-resistant prostate cancer (CRPC), which is a fatal form of PCa that is difficult to confront [6]. Second-generation novel hormonal therapy (NHT), including abiraterone and enzalutamide, has been approved by the Food and Drug Administration (FDA) for the treatment of CRPC and demonstrates good clinical tolerance and overall survival improvement for CRPC patients [4, 5]. However, resistance to abiraterone or enzalutamide remains a painful problem for biologists and clinicians [7, 8].

Studies have identified the biological roles of noncoding RNAs, especially long noncoding RNAs (lncRNAs), in the tumorigenesis of human cancers [9–11]. LncRNAs are a class of ncRNAs that are more than 200 nucleotides in length, and although lncRNAs do not possess protein-coding capability, they play vital roles in modulating protein-coding genes at the transcriptional or posttranscriptional level [10, 11]. Dysregulation of lncRNAs is associated with carcinogenesis [12], metastasis [13] and drug resistance [14]. Mechanistically, lncRNAs have been characterized as sponges with microRNAs (miRNAs) or circular RNAs (circRNAs) and act as competing endogenous RNAs (ceRNAs) to reduce the regulation of their target genes [15]. Recent studies have revealed the ceRNA regulatory network of lncRNAs in a variety of cancer types. In hepatocellular carcinoma, NEAT1 promotes ferroptosis by modulating the miR-362-3p/MIOX axis [16]. In esophageal squamous cell carcinoma, LNC00680 promotes cancer progression through the miR-423-5p/PAL6 axis [17]. In prostate cancer, we previously reported that ZFAS1 competitively sponges miR-15/16/23a and promotes c-Myc expression [18]. However, the functions of lncRNAs in driving PCa progression and modulating drug resistance are not yet fully understood.

The family of small nucleolar RNA host genes (SNHGs) is a lncRNA subgroup that participates in various human biological processes, such as DNA methylation, protein ubiquitination, regulation of gene transcription and translation [19, 20]. SNHG4 belongs to the SNHG family and is located at 5q31.2. The SNHG4 gene consists of exon/intron structures and has nine transcripts. SNHG4 has been reported to play an oncogenic role in many cancer types, including non-small cell lung cancer [21], colorectal cancer [22], and cervical [23] cancer. In addition, SNHG4 was found to be upregulated in prostate cancer tissues and mechanistically promotes prostate cancer cell growth and metastasis by acting as a ceRNA and interacting with miR-377 [24]. To date, although public datasets support that SNHG4 is overexpressed in PCa tissues, limited studies have focused on the underlying mechanisms by which SNHG4 enhances PCa cell survival and resistance to external stimuli, let alone the potential effects of SNHG4 in modulating drug resistance.

Cell senescence is a biological phenomenon that commonly occurs in tumor cells after treatment with targeted therapy or chemotherapy and is termed therapy-induced senescence (TIS) [25, 26]. In senescent cells, proliferation is blocked, and the expression of secretory factors is elevated, which is described as the senescence-associated secretory phenomenon (SASP) [26, 27]. TIS is expected to inhibit tumor growth, as it arrests the progression of the tumor cell cycle; however, whether TIS enhances or restrains cancer development depends on the cellular context and external stimulus [28, 29]. Reportedly, lncRNAs are capable of modulating cell senescence in cancer. One famous molecule is lncRNA nucleotide metabolism regulator (lincNMR), which was originally induced in hepatocellular carcinoma (HCC) [30]. Depletion of lincNMR invokes proliferation defects and triggers senescence in HCC, breast cancer and lung cancer cells. To date, the regulation of cell senescence by lncRNAs in prostate cancer is still poorly understood.

In this study, we identified novel functions of the lncRNA SNHG4 in driving prostate cancer progression and enzalutamide resistance. First, we recognized RRM2 and NUSAP1 as key prognostic markers in PCa by analyzing three GEO datasets. Next, we established a SNHG4/let-7a-5p/RRM2 ceRNA network. We then further studied let-7a-5p targets and overexpressed genes in GEO datasets and verified three other cell cycle controllers, EZH2, TK1 and AURKA, as SNHG4 downstream targets. Gain- and loss-of-function analyses suggested that SNHG4 modulates cell senescence, the cell cycle, cell proliferation, the DNA damage response and enzalutamide resistance in PCa cells. Finally, we validated a regulatory loop of RREB1/SNHG4/let-7a-5p that contributed to the overexpression of SNHG4 in PCa (Figure S1). Overall, our study outlined the significant prognostic value of high SNHG4 expression in prostate cancer and the novel mechanisms by which SNHG4 drives prostate cancer progression and drug resistance.

## Methods

### Tissues

The clinical tissues (benign prostate tissues, prostate cancer tissues and adjacent normal prostate tissues) were freshly collected from thirty patients who were diagnosed with benign prostate hyperplasia or sixty patients who were pathologically diagnosed with prostate cancer and received radical surgical resection. All patients were hospitalized and received surgical treatment at the Urology Department at the First Hospital of China Medical University (Shenyang, China). The study was conducted according to an institutional review board-approved protocol (2012–33) by the Medical Ethics Committee of the First Hospital of China Medical University (authorization number: AF-SOP-07–1.1–01), and written informed consent was obtained from each patient for surgery and research purposes. The clinical pathological sections of normal prostate tissue and prostate cancer tissues were provided by the Department of Pathology at the First Hospital of China Medical University.

### Cell culture

RWPE-1, DU145, 22Rv1, LNCaP and PC-3 cells were purchased from the cell bank of the Chinese Academy of Sciences (Shanghai, China). Enzalutamide-resistant LNCaP cells were generated by serial growth of LNCaP cells under increasing amounts of enzalutamide (1 to 40 µM) for 6 months. These cells were maintained in RPMI 1640 medium supplemented with 10% FBS and antibiotics and regularly tested to ensure that they were mycoplasma-free.

### Antibodies and reagents

Antibodies were purchased from the following companies: RRM2 (ab172476), TK1 (ab76495) and RREB1 (ab64168) were from Abcam (Cambridge, United Kingdom), AURKA (sc373856) was from Santa Cruz Biotechnology (Dallas, TX), EZH2 (#5246) and GAPDH (#5174) were from Cell Signaling Technology (Danvers, MA). Docetaxel (RP-56976) was purchased from MedChemExpress (Shanghai, China).

### Bioinformatic analysis

RNA sequencing data from the GEO database (GSE38241, GSE3325 and GSE104749, https://www.ncbi.nlm.nih.gov/geo/) were used to analyze the differentially expressed genes between normal prostate epithelial and prostate cancer tumor tissues. The analysis of SNHG4, RRM2, NUSAP1, EZH2, AURKA, TK1, NEAT1 and RREB1 was based on The Cancer Genome Atlas (TCGA, https://portal.gdc.cancer.gov/) dataset. Gene expression profiles and clinicopathological characteristics of prostate cancer patients were obtained from the TCGA_PRAD dataset. RNA sequencing data were analyzed using R software (v4.1.3, R core team, March 10, 2022). The DESeq2 package (v1.36.0, Michael Love, March 15th, 2022) was used to normalize gene expression, and read counts were normalized to transcripts per million (TPM). This study complied with the publication guidelines provided by TCGA.

The Encyclopedia of RNA Interactomes (ENCORI, https://starbase.sysu.edu.cn/index.php) was used to predict RRM2-targeting lncRNAs. Tarbase (https://dianalab.e-ce.uth.gr/html/diana/web/index.php?r=tarbasev8) and TargetScan (https://www.targetscan.org/vert_72/) were used to predict miRNA targets. The motif information was obtained from Jaspar (https://jaspar.genereg.net/). Motif-based sequence analysis tools (The MEME Suite, https://meme-suite.org/meme/index.html) were used to predict the upstream TFs targeting SNHG4.

### Animal studies

BALB/c nude mice (4–6 weeks old, 14–16 g) were purchased from Vital River Experimental Animal Technology (Beijing, China) and housed in the Department of Laboratory Animal Science of China Medical University. The study was approved by the Medical Laboratory Animal Welfare and Ethics Committee of China Medical University, and the methods were carried out in accordance with the approved guidelines.

This study includes two animal experiments. To investigate the function of the SNHG4/let-7a/RRM2 axis in prostate cancer cells, control 22Rv1 cells, SNHG4 KD 22Rv1 cells, SNHG4 and let-7a KD 22Rv1 cells or SNHG4 KD with RRM2 OE 22Rv1 cells were separately injected into the flanks of athymic nude mice to establish xenograft tumors (5 mice/group). The length, width, and thickness of the tumors were measured with calipers every 5 days. Forty-five days after injection, experimental mice bearing xenograft tumors were sacrificed, and tumors were removed, weighed and subjected to ISH/IHC staining. Tumor volumes were calculated using the equation (Length × Width2)/2. To investigate the function of SNHG4 in inducing cell resistance to enzalutamide, control LNCaP cells, SNHG4 OE LNCaP cells or SNHG4 MUT LNCaP cells were injected into the flank of BALB/c nude mice (4 weeks, male), and empty vehicle (vehicle without enzalutamide) or enzalutamide was administered once a day via oral gavage at 10 mg/kg (enzalutamide) in 1% carboxymethyl cellulose, 0.1% Tween-80, and 5% DMSO, in each three days after the average volume of the tumors reached 120 mm3 (Group 1: tumor composed of control LNCaP cells, treated by vehicle; Group 2: tumor composed of control LNCaP, treated by enzalutamide; Group 3: tumor composed of SNHG4 OE LNCaP cells, treated by enzalutamide; Group 4: tumor composed of SNHG4 MUT LNCaP cells, treated by enzalutamide, five mice/group). The experimental mice were then sacrificed on the 45th day, and the xenograft tumors were removed, weighed and subjected to IHC staining (Fig. 8h). The length, width, and thickness of tumors were measured with calipers every 5 days, and tumor volumes were calculated using the equation (Length × Width2)/2.

### Statistical analysis

Data are shown as the mean ± SEM from at least three independent experiments. Statistical analyses involved Student’s t test, one-way ANOVA, and Kaplan‒Meier analysis with SPSS 22 (IBM Corp., Armonk, NY) or GraphPad Prism 8.0.1 (GraphPad Software, Inc., La Jolla, CA). Two-tailed Student’s t test was used to determine the difference between two groups of datasets with similar variance, and analysis of variance (ANOVA) was used to determine the difference between more than two groups of datasets. For all statistical analyses, significant differences were labeled as * (*p* < 0.05), ** (*p* < 0.01), or *** (*p* < 0.001). *p* < 0.05 was considered statistically significant.

Additional methods are presented in the Supplemental Methods.

## Results

### RRM2 and NUSAP1 are highly expressed in PCa tumors and significantly correlated with poor clinical outcomes of PCa patients

**Fig. 1:**
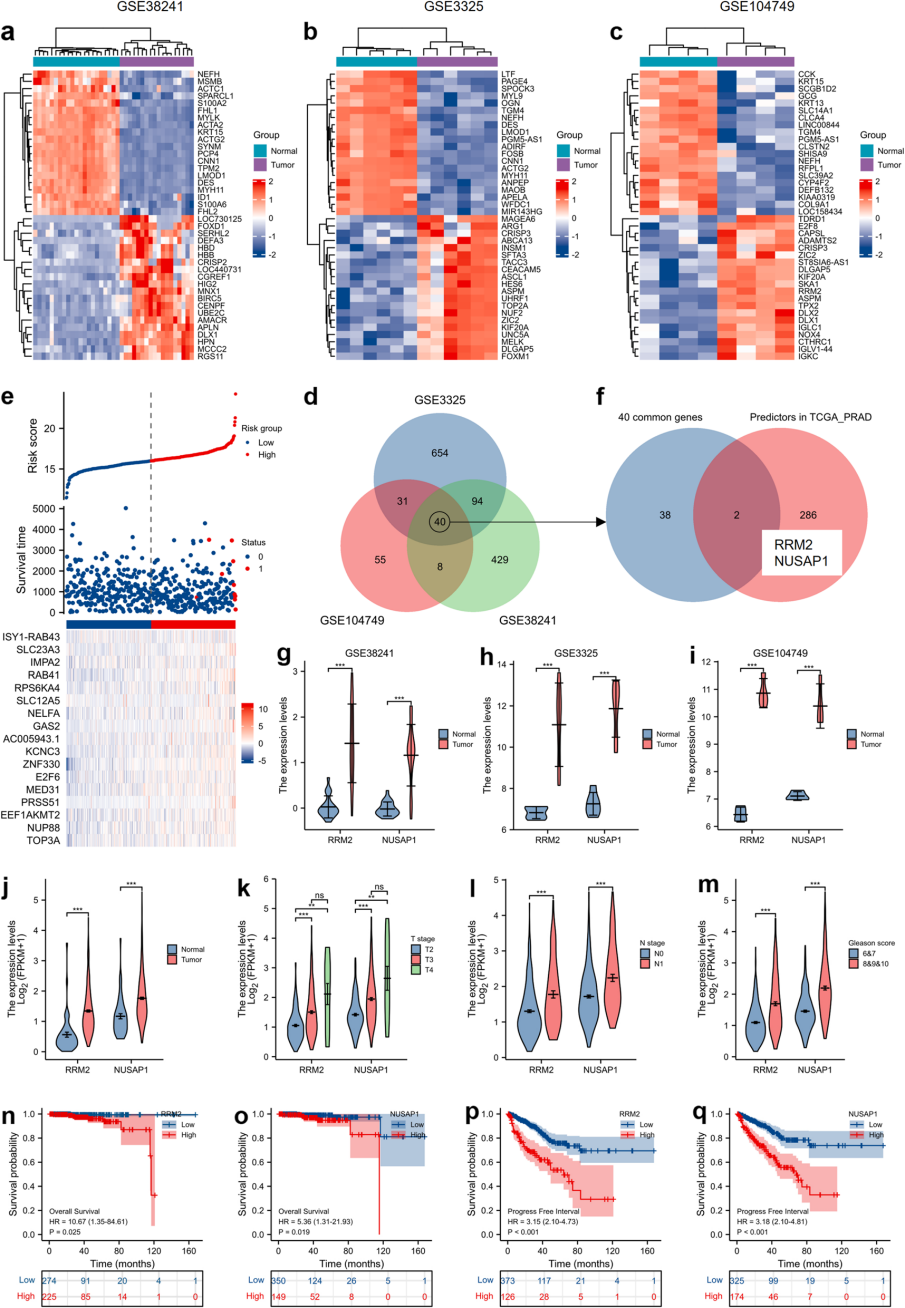
RRM2 and NUSAP1 are highly expressed in PCa tumors and significantly correlated with poor clinical outcomes of PCa patients. Heatmaps show the top 20 upregulated or downregulated genes in GSE38241 (**a**), GSE3325 (**b**) and GSE104749 (**c**). **d** Venn diagram shows the crossed genes among two or three GEO datasets. **e** Diagram shows the risk factors (genes) along with the risk score. Risk factor analysis was performed based on the gene expression profile and overall survival data of patients from the TCGA_PRAD dataset, and 288 genes were identified as risk factors. **f** Venn diagram shows that RRM2 and NUSAP1 were two crossed genes among the 40 common genes from three GEO datasets and 288 risk factors from the TCGA_PRAD dataset. RRM2 and NUSAP1 were more highly expressed in PCa tumor samples than in normal samples in GSE38241 (**g**), GSE3325 (**h**) and GSE104749 (**i**). Analysis based on the TCGA_PRAD dataset shows that RRM2 and NUSAP1 are not only highly expressed in PCa tumors (**j**) but also correlated with tumor progression indexes, including T stage (**k**), N stage (**l**), Gleason score (**m**), overall survival (**n** and **o**) and progression-free survival (**p** and **q**) of PCa patients. ns indicates not significant, * indicates *p* < 0.05, ** indicates *p* < 0.01, *** indicates *p* < 0.001

To explore the potential mechanism driving PCa progression, we first downloaded RNA-seq data from three GEO datasets, GSE38241, GSE3325 and GSE104749. We analyzed the most differentially expressed genes (DEGs) in three GEO datasets, and the top 20 DEGs are shown by heatmaps (Fig. 1a, b and c). We located 40 overexpressed DEGs that commonly appeared in three GEO datasets, and the results are shown in a Venn diagram (Fig. 1d). Next, we analyzed the PRAD_TCGA datasets and located 288 genes that were positively related to the overall survival (OS) of PCa patients (Fig. 1e). We compared the 40 commonly overexpressed genes and OS-related genes, and we concluded that ribonucleotide reductase regulatory subunit M2 (RRM2) and nucleolar and spindle associated protein 1 (NUSAP1) were two common genes across both gene lists (Fig. 1f).

The expression of RRM2 and NUSAP1 in GSE38241, GSE3325 and GSE104749 was analyzed, and we confirmed that RRM2 and NUSAP1 were dramatically overexpressed in PCa tissues (Fig. 1g to i). The expression of RRM2 and NUSAP1 in prostate cancer patients was further verified in PRAD_TCGA datasets. The results suggested that RRM2 and NUSAP1 were significantly overexpressed in PCa tissues (Fig. 1J). In addition, overexpression of RRM2 and NUSAP1 was also observed in patients with advanced T stage (Fig. 1k), lymph node metastasis (Fig. 1l) and advanced Gleason score (Fig. 1m). Next, we analyzed the prognostic values of RRM2 and NUSAP1, and we found that overexpression of RRM2 and NUSAP1 was significantly related to poor overall survival (Fig. 1n and o) and the progression-free interval of PCa patients (Fig. 1p and q). Taken together, our results suggested that RRM2 and NUSAP1 were not only correlated with poor prognosis of PCa patients but also very likely to contribute to the progression of prostate cancer. RRM2 is a key enzyme in deoxyribonucleoside triphosphate (dNTP) biosynthesis and cell senescence [31], and regulation of RRM2 by lncRNA was reported to modulate cell proliferation, senescence and colony formation [30]. Therefore, we aimed to investigate lncRNA-RRM2 regulation in prostate cancer, which may provide novel evidence that key lncRNAs drive PCa progression.

### RRM2 regulates PCa cell viability, the cell cycle and the DNA damage response

**Fig. 2:**
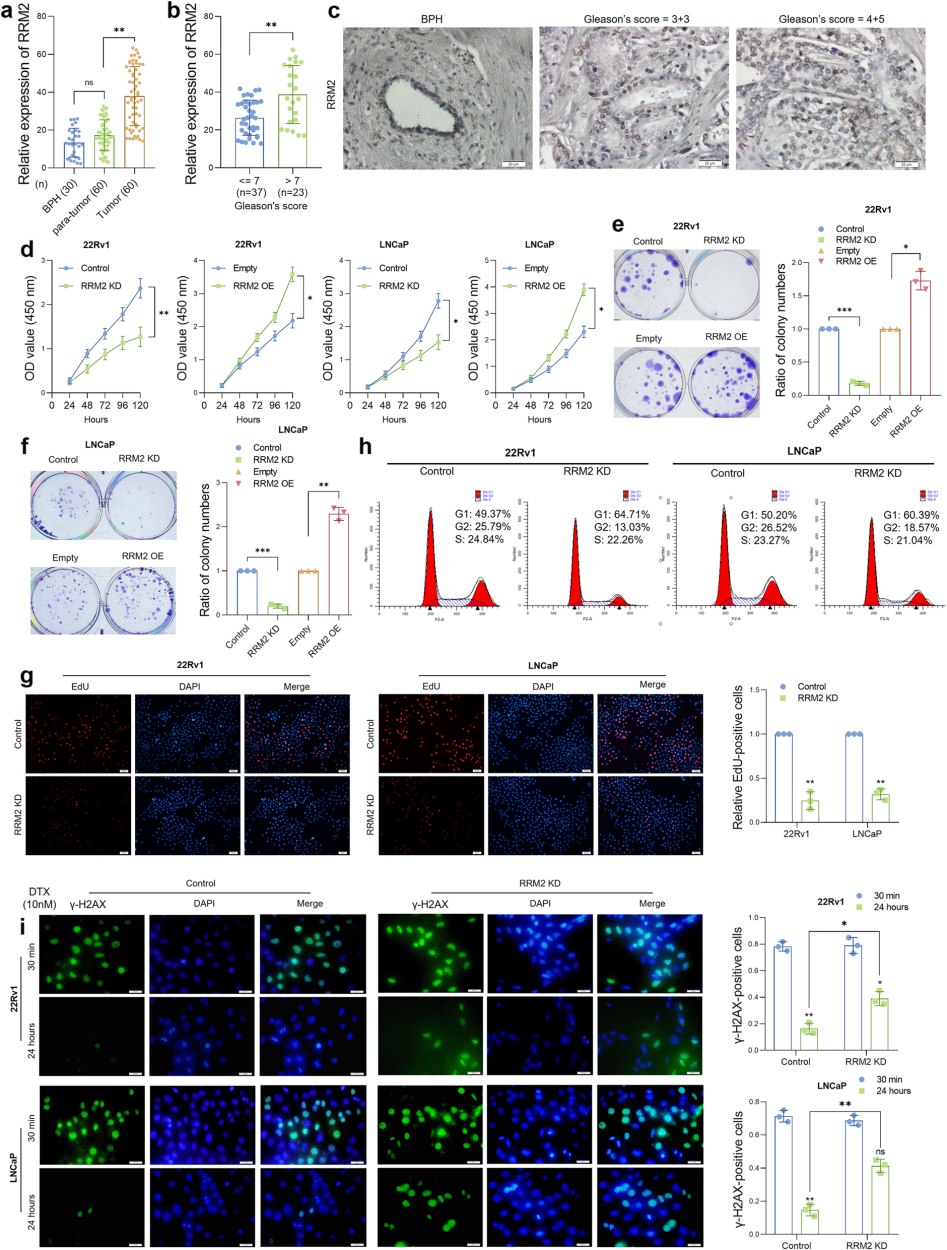
Biological functions of RRM2 in prostate cancer. **a** qRT‒PCR suggested a notable overexpression of RRM2 in PCa tumor tissues. The expression of RRM2 was analyzed in 30 BPH tissues and 60 pairs of PCa and adjacent normal tissue samples by qRT‒PCR. **b** Expression of RRM2 was analyzed and compared between the low Gleason score group (*n* = 37) and the high Gleason score group (*n* = 23). **c** RRM2 is highly expressed in prostate tumor tissues with advanced Gleason scores. Representative images show RRM2 expression in BPH tissues and prostate tumor tissues by IHC staining. The staining intensity of RRM2 was scored as 0 to 5 (0: no staining, 1: very weak staining, 2: weak staining, 3: medium staining, 4: strong staining, 5: very strong staining). Magnification: 200X. **d** RRM2 expression was overexpressed or knocked down in PCa cell lines, and the CCK-8 assay showed the proliferation curve of the cells. **e** and **f** Cell proliferation was assessed by colony formation assay in RRM2 overexpression or knockdown PCa cell lines. **g** RRM knockdown inhibited the proliferation of PCa cells, as determined by EdU staining assay. RRM2 was knocked down in 22Rv1 and LNCaP cells, and after 48 h, the cells were subjected to EdU staining. Numbers of proliferative cells were counted by ImageJ. Red color indicates positively stained cells. Magnification: 100X. **h** RRM2 knockdown induced cell cycle arrest in G1 phase. The cell cycle was measured by FACS in control or RRM2 knockdown 22Rv1 and LNCaP cells. **i** IF staining assay suggested that RRM2 knockdown remarkably induced DBS in PCa cells after DTX treatment. γ-H2AX foci were measured by IF assay in control or RRM2 knockdown PCa cells 30 min or 24 h after DTX treatment (10 nM). Green indicates positively stained cells. Magnification: 200X. ns indicates not significant, * indicates *p* < 0.05, ** indicates *p* < 0.01

Given the prognostic value of RRM2 in prostate cancer, we next examined the expression of RRM2 in our cohort of patients. RRM2 expression was analyzed in normal prostate epithelium tissues from thirty patient diagnosed with benign prostatic hyperplasia (BPH) and sixty pairs of prostate tumor tissues with adjacent normal prostate tissues from sixty patients diagnosed with PCa by quantitative real-time PCR analysis. The clinical information of the PCa patients is shown in Table S1 and S2. The results suggested that RRM2 was highly expressed in tumor tissue samples (Fig. 2a). In addition, we analyzed RRM2 expression in subgroups of PCa patients and found that RRM2 was significantly overexpressed in prostate tumor samples with a high Gleason score (Gleason score > 7, Fig. 2b). We further examined RRM2 expression in clinical tissue specimens by immunohistochemistry staining (IHC). Tissue sections of ten normal prostate epithelium tissues and twenty prostate tumor tissues were collected from the Department of Pathology of our hospital. The results from IHC staining suggested that staining of RRM2 was very weak in normal prostate tissues; in contrast, the heaviest staining of RRM2 was observed in tumor samples with Gleason scores over 7 (Fig. 2c and Figure S2a). Expression of RRM2 in PCa cell lines and normal prostate epithelial cell line RWPE-1 was measured by qRT-PCR (Figure S2b). The results from our cohort further confirmed that RRM2 is overexpressed in PCa tissues and that RRM2 expression correlates with PCa progression.

Next, we investigated the biological functions of RRM2 in prostate cancer cell lines. Two AR-positive PCa cell lines, 22Rv1 and LNCaP, were selected for experiments. RRM2 was overexpressed or depleted by transducing RRM2-overexpressing vectors or siRNAs and validated by qRT‒PCR and western blotting (Figure S2c). Cell Counting Kit-8 (CCK-8) analysis, cell colony formation analysis and 5-ethynyl-2’-deoxyuridine (EdU) staining assays were performed to examine cell survival and proliferation after RRM2 overexpression or depletion. The results suggested that exogenous transfection of RRM2 significantly enhanced cell proliferation, whereas RRM2 knockdown inhibited cell viability and colony formation (Fig. 2d-g). We also found that RRM2 knockdown remarkably induced cell cycle arrest in the G1 stage in 22Rv1 and LNCaP cells (Fig. 2h, Figure S2d). dNTPs are essential for DNA replication and DNA damage repair, and blockade of dNTP biosynthesis induces inhibition of DNA replication, arrest of the cell cycle and DNA damage. To determine whether RRM2 regulates the DNA damage response (DDR) in PCa, we treated RRM2-overexpressing or RRM2-depleted prostate cancer cells with 10 nM Docetaxel (DTX) for 30 min and performed γ-H2AX staining, which is a widely used marker for DNA double-strand breaks (DSBs), at 30 min or 24 h after DTX treatment. At 30 min after treatment, we found similar numbers of γ-H2AX between control and RRM2-depleted PCa cells (Fig. 2i). At 24 h after treatment, the γ-H2AX-positive cells mostly disappeared in control cells, in contrast, some γ-H2AX foci remained in most RRM2-depleted cells. Taken together, we demonstrated that RRM2 is overexpressed in PCa tumors and regulates the cell viability, cell cycle and DDR of prostate cancer cells.

### SNHG4 regulates RRM2 by competitively interacting with let-7a-5p

**Fig. 3:**
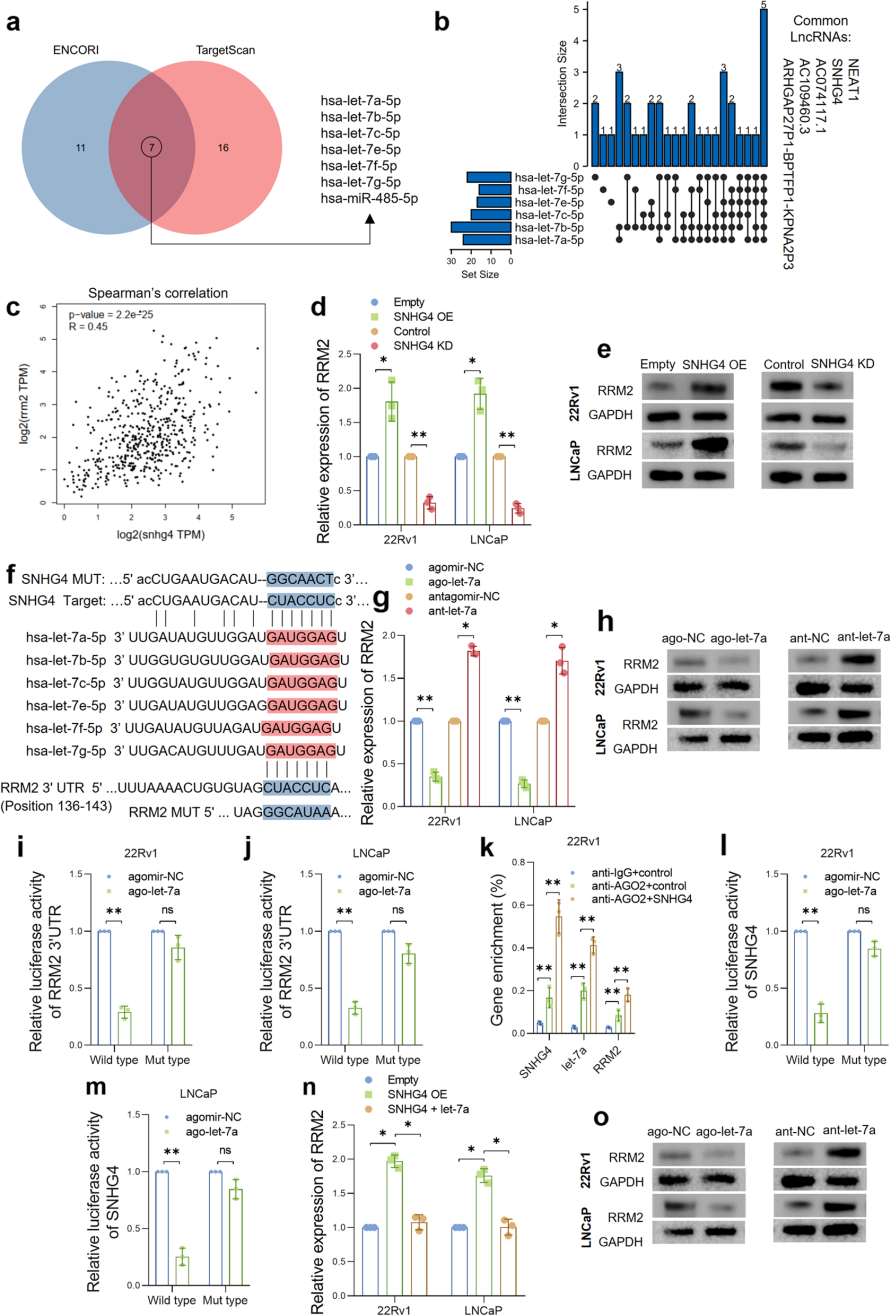
SNHG4 sponges let-7a and regulates RRM2 expression. **a** miRNAs targeting RRM2 were predicted by ENCORI and TargetScan, and seven crossed miRNAs were identified and are shown in a Venn diagram. **b** LncRNAs targeting let-7 miRNAs were predicted by ENCORI, and five lncRNAs were predicted to be their common upstream lncRNAs. **c** Spearman’s correlation coefficient test showed the correlation between the expression of RRM2 and SNHG4 in 499 PCa tissue samples from the TCGA_PRAD dataset (*R* = 0.45, *p* < 0.001). **d** and **e** SNHG4 was overexpressed or knocked down in PCa cells, and RRM2 expression was measured by qRT‒PCR and western blotting. **f** The sequences of predicted binding sites between SNHG4, let-7 miRNAs and RRM2 are shown. **g** and **h** Let-7a-5p was overexpressed or knocked down in PCa cells, and RRM2 expression was analyzed by qRT‒PCR and western blotting. **i** and **j** A dual-luciferase reporter assay was performed to assess the binding between let-7a and the 3’UTR of the RRM2 gene in 22Rv1 and LNCaP cells. **k** RIP assay indicating the enrichment of SNHG4, let-7a and RRM2 in the RNA products pulled down by anti-IgG/anti-AGO antibody before or after SNHG4 overexpression. **l** and **m** A dual-luciferase reporter assay was performed to assess the binding between SNHG4 and let-7a in 22Rv1 and LNCaP cells. Full-length SNHG4 or binding site-mutated SNHG4 was cloned into luciferase plasmids. **n** and **o** Expression of RRM2 was measured in control cells, SNHG4-overexpressing cells or SNHG4 and let-7a double-overexpressing cells by qRT‒PCR and western blot. ns indicates not significant, * indicates *p* < 0.05, ** indicates *p* < 0.01

Given the clinical significance and biological functions of RRM2 in prostate cancer, we next sought to investigate the lncRNA-mediated regulation of RRM2 in PCa. First, two web-based tools, ENCORI and TargetScan, were used to analyze the miRNAs that potentially target the RRM2 gene. The analysis results suggested that there were 18 miRNAs predicted by ENCORI and 23 miRNAs predicted by TargetScan, and only 7 miRNAs were predicted by both tools, namely, hsa-let-7a-5p, hsa-let-7b-5p, hsa-let-7c-5p, hsa-let-7e-5p, hsa-let-7f-5p, hsa-let-7 g-5p and hsa-miR-485-5p (Fig. 3a). Six of the candidates were from the let-7 miRNA family and shared similar seed sequences. Therefore, we chose let-7 family miRNAs for lncRNA prediction. Next, we used ENCORI software to predict potential lncRNAs that may interact with the six let-7 family candidates. Finally, five lncRNAs were expected to interact with all six let-7 miRNAs (Fig. 3b). Among them, SNHG4 and NEAT1 drew our attention for their complex biological functions in cancer regulation. The correlation between the expression of lncRNAs and RRM2 in the PRAD_TCGA dataset was analyzed, and we observed a significant correlation between SNHG4 and RRM2; however, no remarkable correlation was found between NEAT1 and RRM2 (Fig. 3c, Figure S2e).

To investigate whether SNHG4 or NEAT1 regulates RRM2 expression, we overexpressed or knocked down both lncRNAs in PCa cell lines, and RRM2 expression was subsequently examined by qRT‒PCR and western blot assays. The results showed that SNHG4 knockdown significantly decreased RRM2 expression, and RRM2 expression was elevated in line with SNHG4 overexpression (Fig. 3d and e). However, no significant changes in RRM2 expression were observed after ectopic NEAT1 transfection or NEAT1 knockdown (Figure S2f). Therefore, we assumed that SNHG4 regulates RRM2 by interacting with let-7 miRNAs. To validate our hypothesis, binding sites of SNHG4, let-7 miRNAs and the 3’UTR of RRM2 were obtained from TargetScan and ENCORI (Fig. 3f). As there are six let-7 miRNA candidates predicted and all let-7 family members include the same “seed sequence”, we chose let-7a-5p (hereafter referred to as let-7a), which is a representative mature form of let-7 miRNA, to be studied in our research. We first overexpressed let-7a in PCa cell lines (Figure S2g) and examined RRM2 expression by qRT‒PCR and western blot assays. As shown in Fig. 3g and h, the results demonstrated that the let-7a mimic greatly inhibited the expression of RRM2, whereas let-7a silencing significantly increased RRM2 expression. Next, luciferase vectors containing wild-type or mutant RRM2 3’UTR were cotransfected with control or let-7a agomir, and a dual-luciferase reporter assay was performed to assess the interaction between let-7a and the 3’UTR of the RRM2 gene. The results suggested that the let-7a agomir notably inhibited the luciferase activity of the reporter construct containing the wild-type 3’UTR sequence of RRM2 in PCa cell lines (Fig. 3i). To analyze the binding between SNHG4, let-7a and RRM2, we subsequently performed a RIP assay. 22Rv1 cells were transfected with control or SNHG4-overexpressing vectors. At 48 h after transfection, cells were lysed, and RNA was pulled down using negative control anti-IgG or anti-AGO2 antibodies. RNA lysates were then subjected to qRT‒PCR. Figure 3j shows that the levels of let-7a and RRM2 were significantly enriched in AGO2-pulled down RNA products; moreover, SNHG4 overexpression further enriched the contents of let-7a and RRM2. Luciferase reporter constructs containing full-length SNHG4 or mutant SNHG4 (Fig. 3f) were cotransfected with control or let-7a mimics into PCa cells, and dual luciferase assays were performed to validate the interactions between SNHG4 and let-7a. As shown in Fig. 3k, let-7a mimics decreased the transcriptional level of luciferase vectors harboring full-length SNHG4 but had no effects on the transcription of those containing the SNHG4 mutant (predicted let-7a binding site was mutated). To further confirm our hypothesis, SNHG4 was overexpressed in PCa cells with or without let-7a mimics, and RRM2 expression was assessed by qRT‒PCR and western blot analysis. Not surprisingly, RRM2 expression was elevated in accordance with SNHG4 overexpression, and let-7a mimics partially reversed the effect of SNHG4 overexpression on RRM2 expression. These results supported our notion that SNHG4 regulates RRM2 expression by competitively sponging let-7 miRNA.

### SNHG4 plays an oncogenic role in PCa progression

**Fig. 4:**
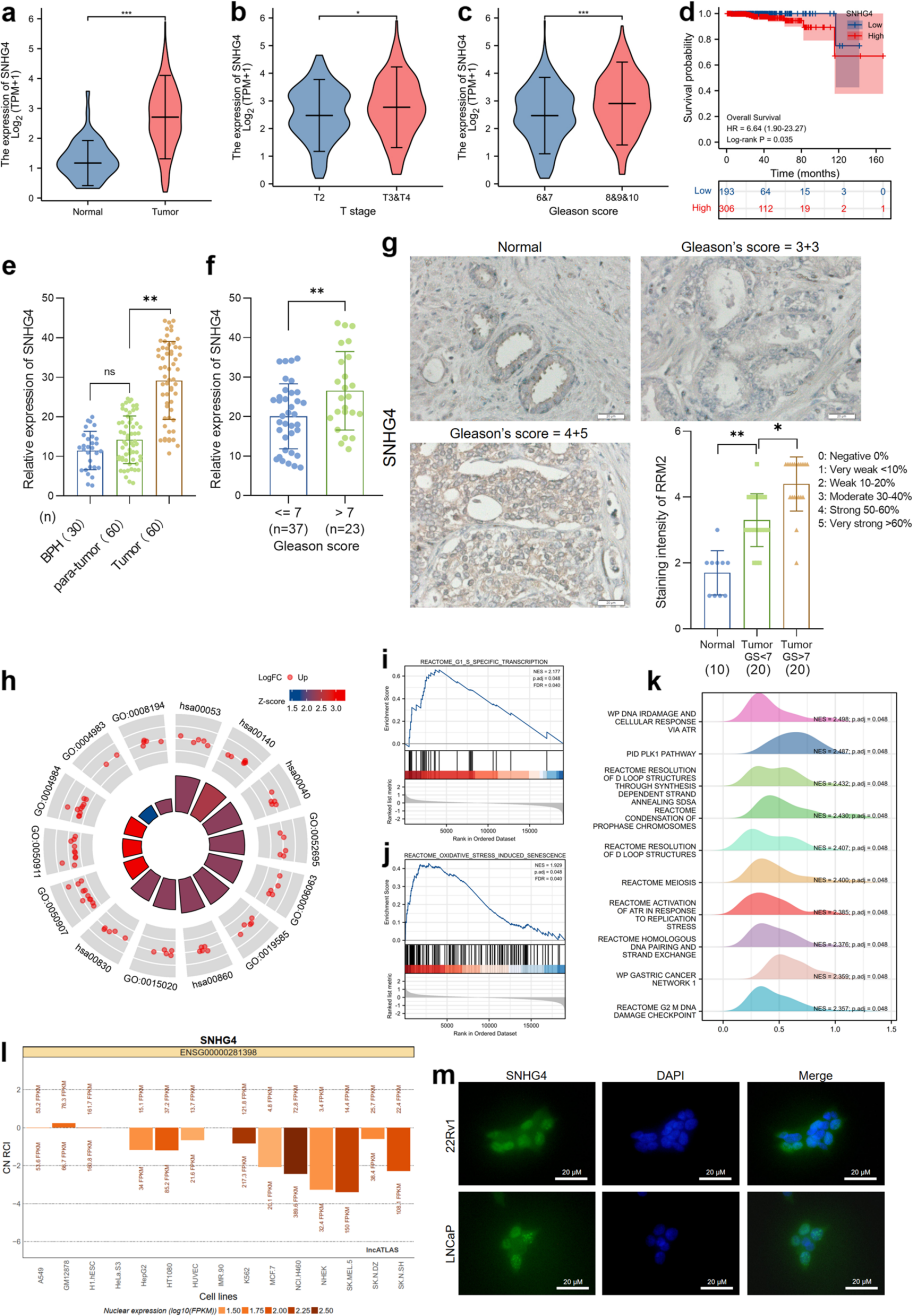
Expression, prognostic value and underlying mechanisms of SNHG4 in prostate cancer. Analysis based on the TCGA_PRAD dataset suggested that SNHG4 is highly expressed in PCa tumor tissues (**a**) and is closely correlated with T stage (**b**) and high Gleason score (**c**) of the tumors. **d** Kaplan‒Meier plot showing that high SNHG4 expression is positively related to poor OS in PCa patients (*p* = 0.035, HR = 6.64). The data were analyzed based on the TCGA_PRAD dataset. **e** Expression of SNHG4 was analyzed in 30 BPH tissues and 60 pairs of PCa and adjacent normal tissue samples by qRT‒PCR. **f** Expression of SNHG4 was analyzed and compared between the low Gleason score group (*n* = 37) and the high Gleason score group (*n* = 23). **g** Representative images showing SNHG4 expression in BPH tissues, PCa tissues with Gleason score < 7 and PCa tissues with Gleason score > 7 measured by ISH staining. Magnification: 200X. **h** Chord diagram indicates the enriched biological processes and pathways of SNHG4 in prostate cancer. Gene set enrichment analysis suggested that SNHG4-correlated genes were enriched in biological processes such as “G1_S_Specific_Transcription” (**i**) and “Oxidative_Stress_Induced_Senescence” (**j**). **k** Mountain diagram shows some key biological processes in which SNHG4-correlated genes were enriched in prostate cancer. **l** Subcellular locations of SNHG4 in different cell lines were predicted by LncATLAS tools. The localization of lncRNAs was calculated as the cytoplasmic/nuclear relative concentration index (CN RCI). **m** Representative images of the subcellular location of SNHG4 in 22Rv1 and LNCaP cells by immunostaining. Scale bar = 20 μM. ns indicates not significant, * indicates *p* < 0.05, ** indicates *p* < 0.01, *** indicates *p* < 0.001

As we have identified SNHG4-let-7 miRNA-RRM2 regulation in prostate cancer, we then sought to evaluate the prognostic role and biological functions of SNHG4 in PCa. To examine the role of SNHG4 in prostate cancer, we used TCGA databases to analyze the expression patterns of SNHG4 between the normal group (*n* = 52) and tumor group (*n* = 499) and subgroups of PCa. The results suggested that SNHG4 is highly expressed in the PCa tumor group (Fig. 4a). In addition, SNHG4 was found to be significantly overexpressed in tumors with advanced T stage (T3&T4 versus T2, Fig. 4b) and Gleason score (> 7 versus <  = 7, Fig. 4c). We further analyzed the prognostic role of SNHG4 in prostate cancer, and survival analysis suggested that high SNHG4 expression was correlated with poor OS (HR = 6.64, *p* = 0.035, Fig. 4d) and PFI (HR = 2.09, *p* < 0.001, Figure S2h) in PCa patients. Expression of SNHG4 in PCa cell lines and normal prostate epithelial cell line RWPE-1 was measured by qRT-PCR (Figure S2i). Next, we measured SNHG4 expression in our cohort of sixty PCa patients and thirty BPH patients. The results showed that SNHG4 expression was elevated in tumor tissues (*n* = 60) compared with adjacent normal tissues (*n* = 60) and normal prostate tissues from BPH patients (*n* = 30) (Fig. 4e). In addition, we observed significantly high SNHG4 expression in tumor samples with high Gleason scores (Fig. 4f). Subsequently, in situ hybridization (ISH) staining was performed to assess SNHG4 expression in clinical samples. As shown in Fig. 4g, according to the qRT‒PCR results, SNHG4 was highly expressed in tumor tissues with a high Gleason score. The results from TCGA databases and our cohort supported an oncogenic role of SNHG4 in prostate cancer progression.

To identify the putative mechanisms by which SNHG4 participates in the progression of prostate cancer, GO/KEGG analysis and gene set enrichment analysis were performed. The results indicated that SNHG4 was enriched in key terms such as “cellular glucuronidation” (GO: 0052695), “uronic acid metabolic process” (GO: 0006063) and “glucuronate metabolic process” (GO: 0019585), and pathways such as “Ascorbate and aldarate metabolism” (hsa00053) and “Steroid hormone biosynthesis” (hsa00140) were correlated with high expression of SNHG4 (Fig. 4h). GSEA showed that SNHG4 was enriched in pathways such as “G1 S-specific transcription” (Fig. 4i), “oxidative stress-induced senescence” (Fig. 4j) and “cell cycle” (Figure S2j), which suggested that SNHG4 may facilitate cell proliferation by regulating the cell cycle and senescence. In addition, enrichment analysis (Fig. 4k) demonstrated that SNHG4 was positively correlated with pathways of “DNA IR damage and cellular response via ATR”, “meiosis”, “G2 M DNA damage checkpoint”, etc., indicating a potential role of SNHG4 in the DNA damage response and cell meiosis. It was reported that the subcellular locations of SNHG family members were mostly in the nucleus and cytoplasm. Data from lncATLAS suggested that SNHG4 was located in the nucleus in most cell lines (Fig. 4l). To evaluate the location of SNHG4 in prostate cancer cells, we performed an immunofluorescence assay using 22Rv1 and LNCaP cells. The results indicated that SNHG4 was located in both the nucleus and cytoplasm (Fig. 4m).

### The cell cycle regulators EZH2/AURKA/TK1 are targeted by let-7 miRNAs and regulated by SNHG4 via a ceRNA network

**Fig. 5:**
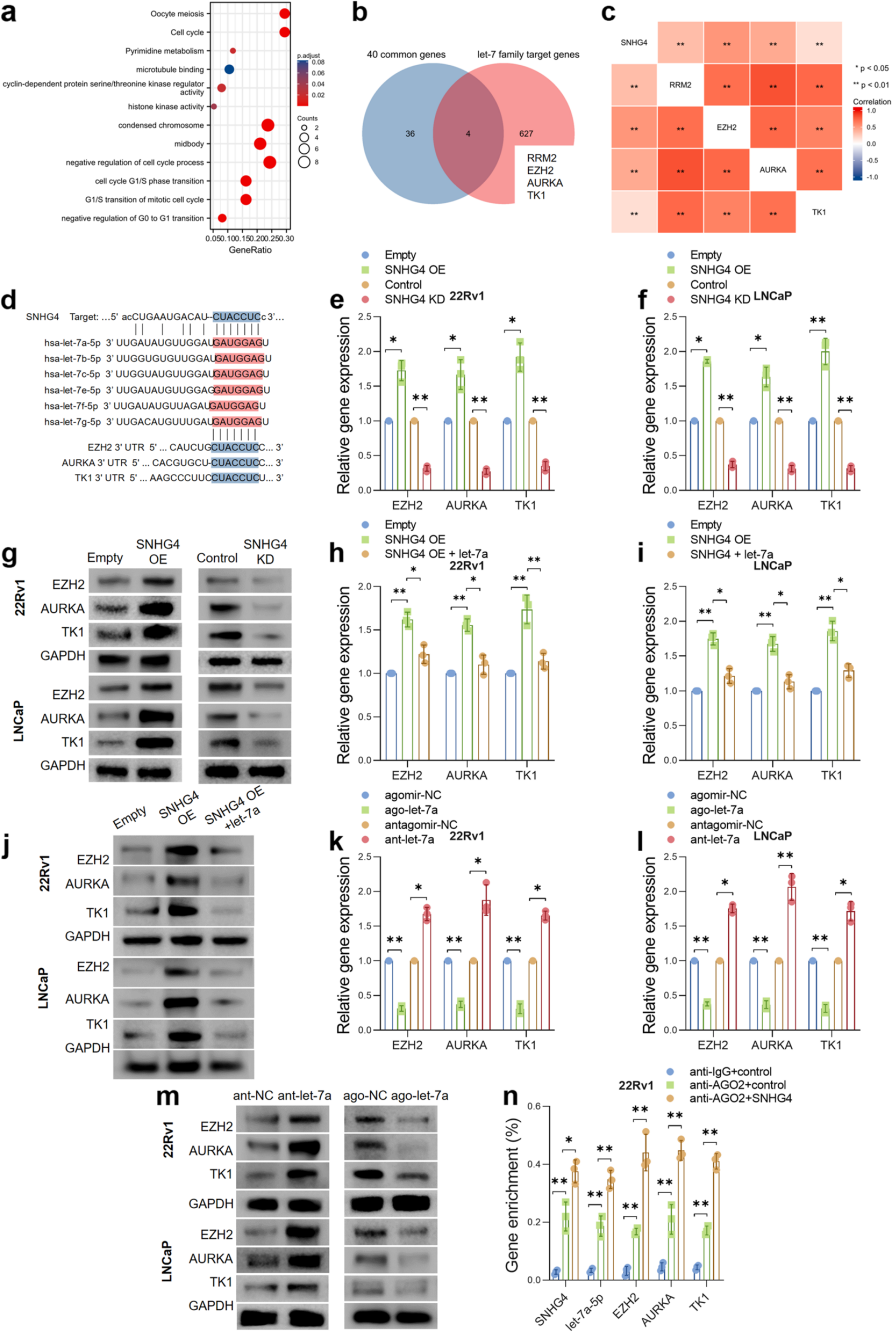
The cell cycle regulators EZH2/AURKA/TK1 are targeted by let-7 miRNAs and regulated by SNHG4 via a ceRNA network. **a** GO/KEGG analysis showed the enriched biological terms of the 40 genes that were overexpressed across three GEO datasets. **b** Venn diagram showing that RRM2, EZH2, AURAK and TK1 were overexpressed across the three GEO datasets and potential target genes of let-7 miRNAs. **c** SNHG4, RRM2, EZH2, AURKA and TK1 were coexpressed genes in PCa tumor tissues, and the data were analyzed based on the TCGA_PRAD dataset. **d** The sequences of predicted binding sites between SNHG4, let-7 miRNAs and EZH2, AURKA and TK1 are shown. **e**, **f** and **g** SNHG4 was overexpressed or knocked down in 22Rv1 and LNCaP cells, and the expression of EZH2, AURKA and TK1 was measured by qRT‒PCR and western blotting. **h**, **i** and **j** Expression of EZH2, AURKA and TK1 was measured in control cells, SNHG4-overexpressing cells or SNHG4 and let-7a double-overexpressing cells by qRT‒PCR and western blot. **k**, **l** and **m** Expression of EZH2, AURKA and TK1 was measured in control/let-7a-overexpressing or scramble/let-7a-knockdown PCa cells by qRT‒PCR and western blot. **n** RIP assay indicating the enrichment of SNHG4, let-7a, EZH2, AURKA and TK1 in the RNA products pulled down by anti-IgG/anti-AGO antibody before or after SNHG4 overexpression. ns indicates not significant, * indicates *p* < 0.05, ** indicates *p* < 0.01

To further explore the underlying mechanisms of SNHG4 in prostate cancer progression, we again focused on the 40 genes that were commonly overexpressed in GSE38241, GSE3325 and GSE104749. GO/KEGG analysis was performed to evaluate the biological terms or pathways in which the 40 genes were enriched. As shown in Fig. 5a, we found that the overexpressed genes were mostly enriched in pathways such as “nuclear division” (GO:0000280), “meiotic cell cycle” (GO:0051321), “negative regulation of mitotic cell cycle” (GO:0045930), “regulation of G2/M transition of mitotic cell cycle” (GO:0010389), and “pyrimidine metabolism” (hsa00240). Next, we predicted the let-7 miRNA target genes by TargetScan and obtained a list of 631 target genes. We compared the 40 common genes with the 631 target genes and found that there were 4 genes across two gene lists, namely, RRM2, EZH2, AURKA and TK1 (Fig. 5b). Interestingly, in line with RRM2, EZH2, AURKA and TK1 are key enzymes that control the cell cycle and senescence. We assumed that SNHG4 facilitates prostate cancer cell proliferation, the cell cycle and senescence by regulating RRM2, EZH2, AURKA and TK1.

As we have identified that SNHG4 regulates RRM2 via let-7 miRNA in PCa cell lines, we then sought to validate whether EZH2, AURKA and TK1 were also downstream targets of SNHG4. Data from TCGA databases suggested that SNHG4 expression was significantly correlated with the expression of RRM2, EZH2, AURKA and TK1 in prostate tumor samples (*p* < 0.01, Fig. 5c). Binding sites between SNHG4, let-7 miRNAs and EZH2, AURKA and TK1 were predicted by TargetScan and ENCORI (Fig. 5d). qRT‒PCR and western blot analysis indicated that the expression of EZH2, AURKA and TK1 in PCa cell lines was changed in accordance with SNHG4 overexpression or knockdown (Fig. 5e to g). Furthermore, restoration of let-7a was capable of reducing the expression of EZH2, AURKA and TK1, which were induced by SNHG4 overexpression (Fig. 5h to j). Next, we overexpressed or depleted let-7a in PCa cell lines and measured the expression of EZH2, AURKA and TK1. The results demonstrated that let-7a overexpression dramatically inhibited the transcription and translation of EZH2, AURKA and TK1, whereas let-7a knockdown showed the opposite effect on the expression of EZH2, AURKA and TK1 (Fig. 5k to m). RIP assays were performed to validate the interactions between SNHG4, let-7a and EZH2, AURKA and TK1. As described before, control 22Rv1 cells or SNHG4-overexpressing 22Rv1 cells were lysed, and RNA was pulled down using negative control anti-IgG or anti-AGO2 antibodies. Figure 5n shows that the levels of let-7a and EZH2/AURKA/TK1 were significantly enriched in anti-AGO2-pulled down RNA products; additionally, the levels of let-7a and EZH2/AURKA/TK1 were further enriched after SNHG4 overexpression. To validate our findings in clinical samples, we performed ISH/IHC staining in thirty prostate cancer tumor tissue sections, and the sections were from PCa patients with a relatively similar Gleason score (from 6 to 7). The staining intensity of the sample was rated by two pathological experts from 0 (negative) to 5 (very strong). Staining intensity from 1 to 3 was identified as low expression, whereas intensity from 4–5 was identified as high expression. The results suggested that high expression of SNHG4 was correlated with RRM2/EZH2/AURKA/TK1 overexpression in prostate cancer tissue specimens (Figure S2k and S2l). These data suggested that SNHG4 regulates EZH2, AUKRA and TK1 through a let-7a-mediated ceRNA network.

### Depletion of SNHG4, RRM2, EZH2, AURKA or TK1 suppresses cell viability and induces senescence and SASP in PCa cells

**Fig. 6:**
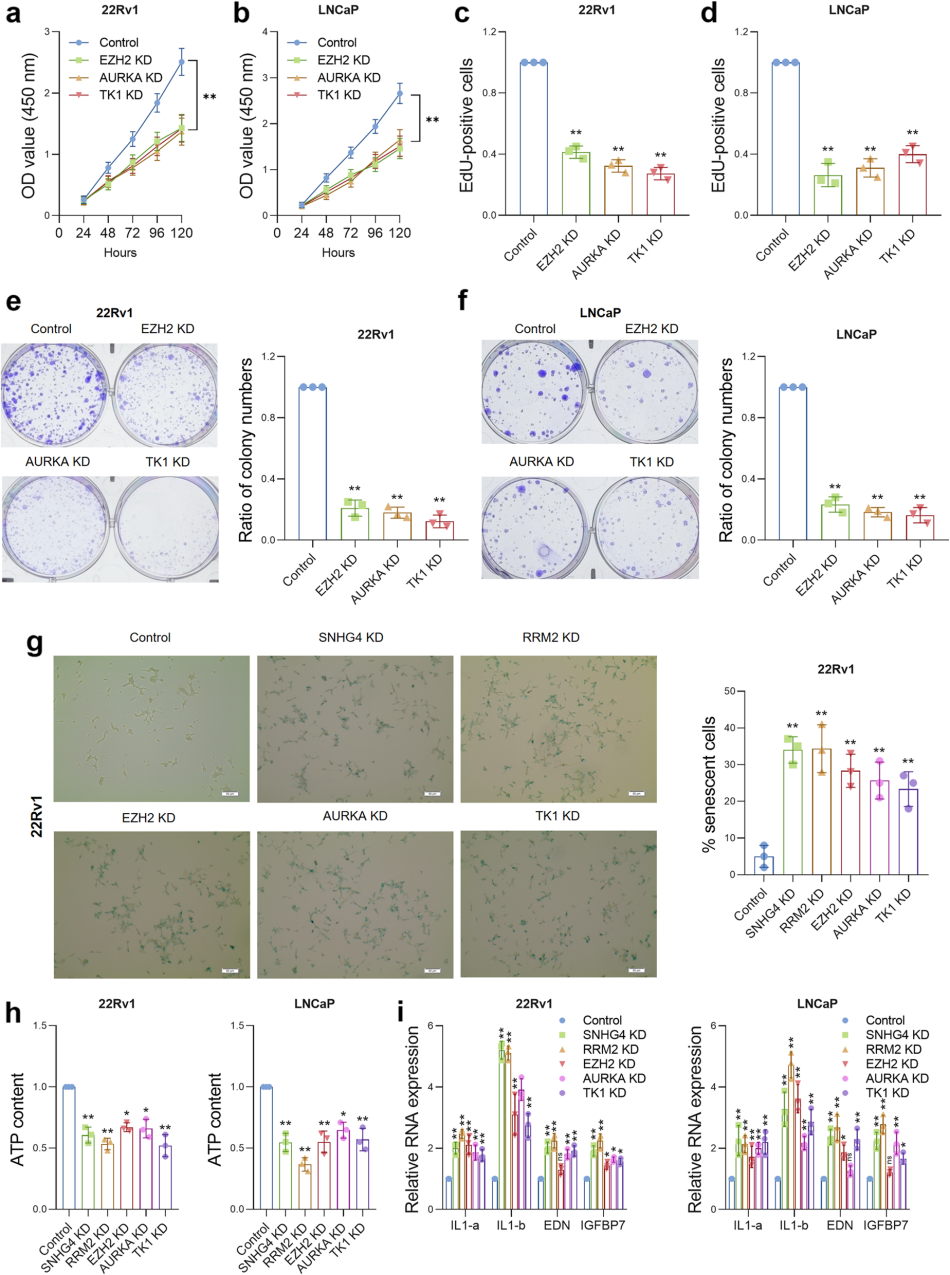
Depletion of SNHG4, RRM2, EZH2, AURKA or TK1 suppresses cell viability and induces senescence and SASP in PCa cells. **a** and **b** EZH2, AURKA or TK1 expression was knocked down in PCa cell lines, and the CCK-8 assay showed the proliferation curve of the cells. **c** and **d** EZH2, AURKA or TK1 was knocked down in 22Rv1 and LNCaP cells, and after 48 h, the cells were subjected to EdU staining. Numbers of proliferative cells were counted by ImageJ. **e** and **f** Cell proliferation was assessed by colony formation assay in EZH2, AURKA or TK1 expression knockdown PCa cell lines. **g** Representative images of 22Rv1 cells after SA-β-Gal staining. SNHG4, RRM2, EZH2, AURKA or TK1 was depleted in 22Rv1 cells, and cell senescence was measured by SA-β-Gal assay. Blue color indicated the positively stained cells, which were senescent cells. Magnification: 200X. **h** The ATP content was measured in SNHG4-, RRM2-, EZH2-, AURKA- or TK1-depleted PCa cells. **i** Expression of SASP markers was measured in SNHG4-, RRM2-, EZH2-, AURKA- or TK1-depleted PCa cells by qRT‒PCR. ns indicates not significant, * indicates *p* < 0.05, ** indicates *p* < 0.01

The phenotype of cell senescence can be induced by antitumor therapies such as chemotherapy or targeted therapy and is closely related to sensitivity to antitumor therapy [28]. Studies have revealed that knockdown of RRM2 [30], EZH2 [32], AURKA [33] or TK1 [30] could cause cell senescence. Thus, we aimed to investigate whether depletion of SNHG4, RRM2, EZH2, AURKA or TK1 can cause prostate cancer cell senescence and affect cell viability. The expression of EZH2, AURKA or TK1 was knocked down in PCa cells (Figure S3a), and CCK-8 assays (Fig. 6a and b), EdU assays (Fig. 6c, d and S3b) and colony formation assays (Fig. 6e and f) were performed to assess cell viability. The results indicated that depletion of the above factors significantly inhibited the proliferative capacity of 22Rv1 and LNCaP cells. Next, a senescence-associated β-Gal (SA-β-Gal) assay was performed to evaluate the cell senescence phenotype after knockdown of the above genes. As shown in Fig. 6g and Figure S4a, β-Gal-positive blue cells were significantly higher after the depletion of either SNHG4, RRM2, EZH2, AURKA or TK1. ATP assays showed that knockdown of SNHG4, RRM2, EZH2, AURKA or TK1 significantly reduced the ATP content in PCa cells (Fig. 6h). Furthermore, the senescence-associated secretory phenotype (SASP) markers IL-1a, IL-1b, EDN and IGFBP7 were determined by qRT‒PCR, and the results indicated that the senescence-associated secretory phenotype was significantly induced after knockdown of SNHG4, RRM2, EZH2, AURKA or TK1 in two cell lines (Fig. 6i).

### The SNHG4/let-7a/RRM2 axis modulates the cell viability, cell cycle and DNA damage response of PCa cells.

Given the biological functions of RRM2 in cell viability and cycle control and the regulatory mechanisms of SNHG4 on RRM2 expression, we next attempted to determine whether SNHG4 facilitates phenotypes of PCa cell lines through the let-7a/RRM2 axis. PCa cells were transfected with empty vectors or shRNA against SNHG4, and the expression of let-7a or RRM2 was depleted or restored in SNHG4-overexpressing cells by cotransfecting let-7a antagomir or lentivirus vectors encoding RRM2, respectively. We found that knockdown of SNHG4 significantly decreased RRM2 expression, whereas in SNHG4/let-7a double knockdown cells and RRM2 reoverexpressed SNHG4-knockdown cells, we observed a notable restoration of RRM2 expression (Figure S4b—S4d).

**Fig. 7:**
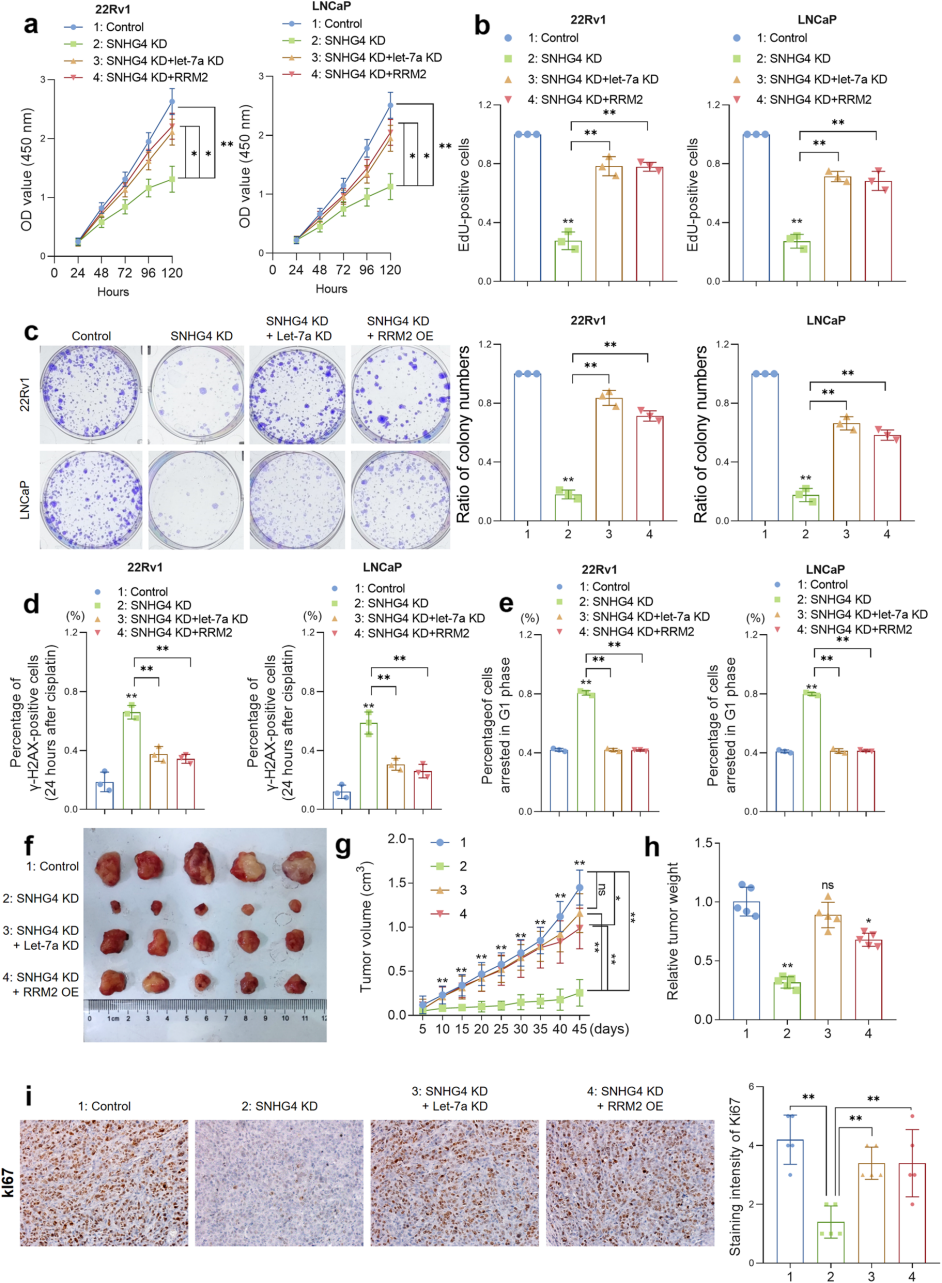
The SNHG4/let-7a/RRM2 axis modulates the cell viability, cell cycle and DNA damage response of PCa cells. CCK-8 assay (**a**), EdU assay (**b**) and colony formation assay (**c**) were performed to measure cell proliferation in control cells, SNHG4-depleted cells, SNHG4 and let-7a double-depleted cells or SNHG4-depleted with RRM2 reoverexpressed cells. **d** γ-H2AX foci were measured by immunostaining assay in four groups of experimental cells 24 h after DTX treatment (10 nM). **e** The cell cycle was measured by FACS in four groups of experimental cells 48 h after treatment. **f** In vivo tumor lumps removed from four groups of CDX mice. **g** The tumor growth curves for in vivo tumor volumes. **h** The mean tumor weight of each group. **i** SNHG4 knockdown reduced Ki67 expression in xenograft tumors, whereas simultaneous let-7a knockdown or RRM2 reoverexpression rescued Ki67 expression, as shown by IHC staining. Magnification: 200X. ns indicates not significant, * indicates *p* < 0.05, ** indicates *p* < 0.01

After the engineered PCa cells were established, cell viability was measured by CCK-8, EdU and colony formation assays, and the cell cycle was analyzed by FACS analysis. In addition, γ-H2AX staining was performed to assess the capability of DNA damage repair. The results suggested that SNHG4 knockdown dramatically inhibited cell proliferation (Fig. 7a, b and S4e) and colony formation (Fig. 7c) of PCa cells; in contrast, restoration of RRM2 by either let-7a knockdown or RRM2 reoverexpression rescued the hampered cell viability. As shown in Fig. 7d and Figure S5a, SNHG4 KD cells revealed the strongest staining of γ-H2AX 24 h after treatment with DTX, whereas restoration of RRM2 significantly decreased the γ-H2AX-positive cell numbers. Moreover, the number of cells in G1 phase was notably increased in SNHG4 KD cells, and recovery of RRM2 expression considerably diminished the number of G1 phase-arrested cells (Fig. 7e and Figure S5b). Subsequently, we sought to validate our findings in a cell-derived xenograft (CDX) model. Four groups of engineered cells were injected into the flanks of BALB/c nude mice (5 mice/group), and the length and width of xenograft tumors were measured every 5 days. Forty-five days after injection, experimental mice bearing xenograft tumors were sacrificed, and tumors were removed, weighed and subjected to ISH/IHC staining. The results demonstrated that SNHG4 KD notably inhibited xenograft tumor growth in volume and weight, whereas RRM2 restoration partially recovered the growth of xenograft tumors (Fig. 7f to h). Finally, IHC staining suggested that the Ki67 level was in line with the size of the tumors (Fig. 7i). Taken together, the above data indicated that SNHG4 modulates the cell proliferation, cell cycle and DNA damage response of PCa cells by regulating RRM2 via a let-7 miRNA-mediated ceRNA network, and the expression of RRM2, EZH2, AURKA and TK1 was altered in accordance with SNHG4 knockdown.

### SNHG4 enhances prostate cancer cell resistance to enzalutamide through the let-7 miRNAs-mediated ceRNA network

**Fig. 8:**
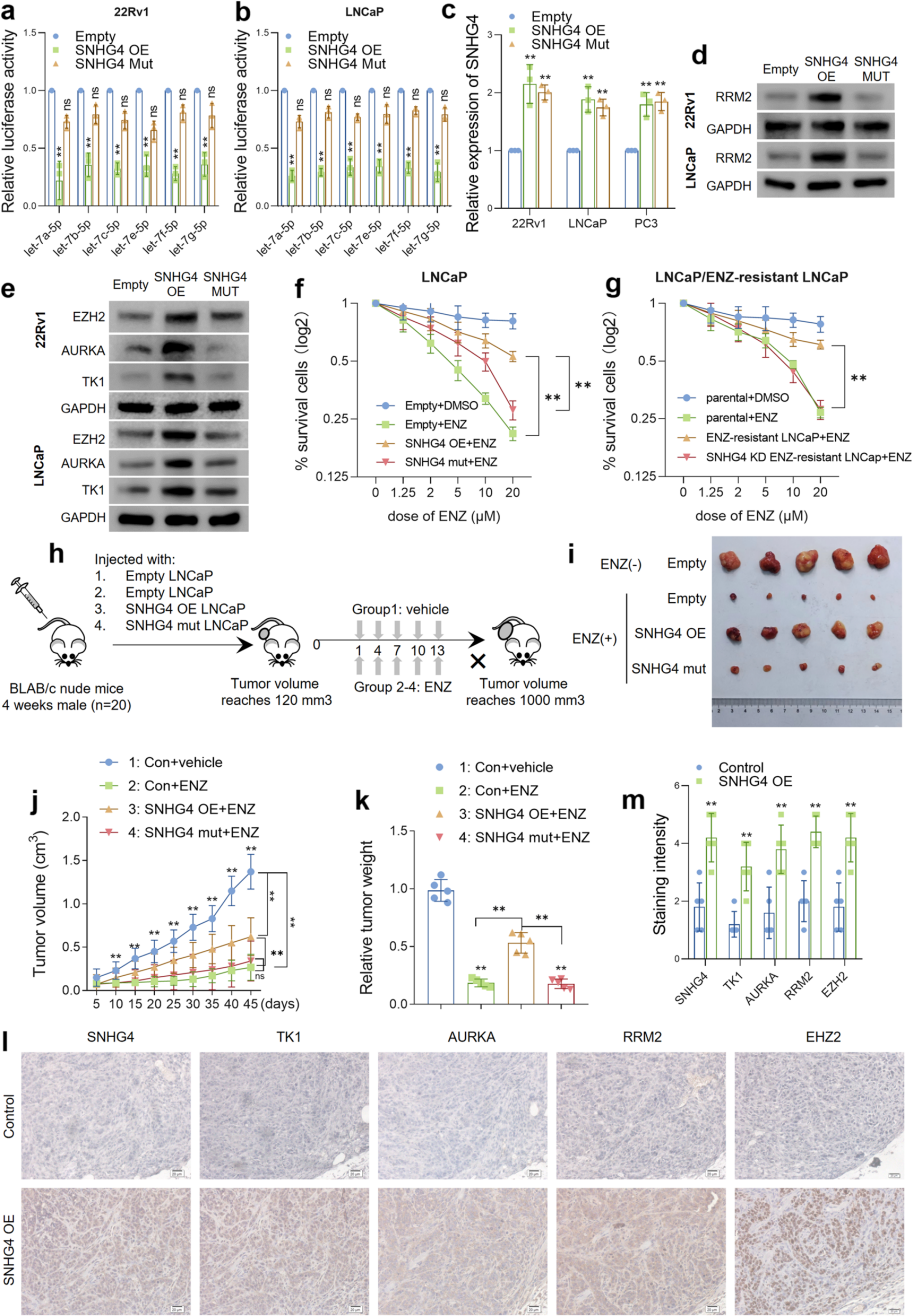
SNHG4 enhances cell resistance to enzalutamide in vitro and in vivo. **a** and **b** A dual-luciferase assay was performed to assess the interactions between let-7 miRNAs and luciferase vectors containing wild-type or mutant SNHG4 in 22Rv1 and LNCaP cells. **c** qRT‒PCR confirmed that overexpressing plasmids containing either wild-type or mutant SNHG4 were capable of inducing the overexpression of the SNHG4 gene in PCa cells. **d** and **e** Western blot analysis was performed to measure the expression of RRM2, EZH2, AURKA and TK1 in response to overexpression of wild-type or mutant SNHG4. **f** Control LNCaP cells and engineered LNCaP cells (overexpression of wild-type or mutant SNHG4) were treated with DMSO or increasing doses of enzalutamide for 24 h, and the cell survival rate was assessed by CCK-8 analysis. **g** The cell survival curve showed that compared to parental LNCaP cells, enzalutamide-resistant LNCaP cells demonstrated significant resistance to enzalutamide treatment; however, knockdown of SNHG4 notably weakened the enza-resistant ability. **h** Diagram showing the flow of animal experiments. **i** In vivo tumor lumps removed from four groups of CDX mice. **j** The tumor growth curves for in vivo tumor volumes. **k** The mean tumor weight of each group. **i** Expression of SNHG4, RRM2, EZH2, AURKA or TK1 was measured in xenograft tumors composed of control or SNHG4-overexpressing LNCaP cells by ISH/IHC staining. Magnification: 200X. **m** The staining intensity of each gene/protein in xenograft tumors composed of control or SNHG4-overexpressing LNCaP cells was calculated and compared. ns indicates not significant, * indicates *p* < 0.05, ** indicates *p* < 0.01

To further confirm that SNHG4 regulates the expression of RRM2, EZH2, AURKA and TK1 by sponging let-7 miRNAs, we constructed reporter plasmids harboring wild-type SNHG4 or the full-length SNHG4 gene with the let-7 binding site (CUACCUC) mutated, and a dual-luciferase reporter assay was performed to assess the interactions between SNHG4 and let-7 miRNAs. As shown in Fig. 8a and b, cotransfection of let-7 miRNAs significantly reduced the transcription of reporter plasmids containing wild-type SNHG4; however, no remarkable changes in luciferase activities were observed when let-7 mimics were cotransfected with the mutant type of SNHG4 into the cells. Next, we constructed an overexpressing plasmid harboring the mutant type of SNHG4, and the transfection efficiency was examined in PCa cells. The qRT‒PCR results suggested that both the wild-type and mutant types of SNHG4 were capable of elevating the expression of SNHG4 in PCa cells (Fig. 8c). The expression of RRM2, EZH2, AURKA and TK1 was detected by western blot analysis, and the results suggested that wild-type SNHG4-overexpressing plasmids notably upregulated the translational expression of the above genes. In contrast, overexpressing plasmids containing mutant SNHG4 failed to alter the expression of RRM2, EZH2, AURKA or TK1 in PCa cells (Fig. 8d and e). These results further confirmed that SNHG4 regulated RRM2, EZH2, AURKA and TK1 via a let-7 miRNA-mediated ceRNA network.

Given the biological functions of SNHG4 in PCa cells, which suppress cell senescence, promote the cell cycle and enhance cell viability and DNA damage repair, we then sought to determine whether SNHG4 facilitates the drug resistance of PCa cells. Enzalutamide is the first-line drug for CRPC, and resistance to enzalutamide is closely related to poor prognosis; hence, enzalutamide remains a painful problem for clinicians [8]. Therefore, we next asked whether SNHG4 affects cell resistance to enzalutamide. We transfected LNCaP cells with empty vector, wild-type SNHG4-overexpressing plasmids or plasmids carrying mutant SNHG4 sequences, and the engineered cells were treated with DMSO or increasing doses of enzalutamide. After 24 h, CCK-8 assays were performed, and the survival rates were calculated (Fig. 8f). We found that the number of LNCaP cells was significantly decreased in accordance with treatment with increasing doses of enzalutamide. Surprisingly, SNHG4 overexpression partially neutralized the cytotoxicity of enzalutamide; in contrast, the mutant type of SNHG4 was not capable of inducing cellular resistance to enzalutamide. Next, we knocked down SNHG4 in ENZ-resistant LNCaP cells, which were constructed as described in our previous study [34]. We confirmed that knockdown of SNHG4 hampered the cellular resistance to enzalutamide of the ENZ-resistant LNCaP cells (Fig. 8g). Subsequently, we built a xenograft model to validate our hypothesis. Control LNCaP cells, SNHG4 OE LNCaP cells or SNHG4 MUT LNCaP cells were injected into the flank of BALB/c nude mice (4 weeks, male), and vehicle or enzalutamide was administered (see materials and methods for detail) in each three days after the average volume of the tumors reached 120 mm^3^. The experimental mice were then sacrificed on the 45th day, and the xenograft tumors were removed, weighed and subjected to IHC staining (Fig. 8h). The results from the xenograft model further supported our notion that enzalutamide dramatically inhibited the growth of the tumors (Fig. 8i and j) and decreased the weight of the tumors (Fig. 8k). Moreover, the expression of SNHG4, TK1, AURKA, RRM2 and EZH2 in the tumor tissues was found to be significantly elevated after SNHG4 overexpression (Fig. 8l and m). Finally, Ki67 expression was measured in each group, and the results supported that the Ki67 level was markedly decreased after enzalutamide treatment, and SNHG4 overexpression partially rescued the Ki67 level (Figure S5f). Taken together, SNHG4 overexpression notably enhanced cell resistance to enzalutamide, which was dependent on the regulation of TK1, AURKA, RRM2 and EZH2 by SNHG4.

### SNHG4 is transcriptionally regulated by RREB1 and regulates RREB1 expression through a let-7 miRNA-mediated positive feedback loop

**Fig. 9:**
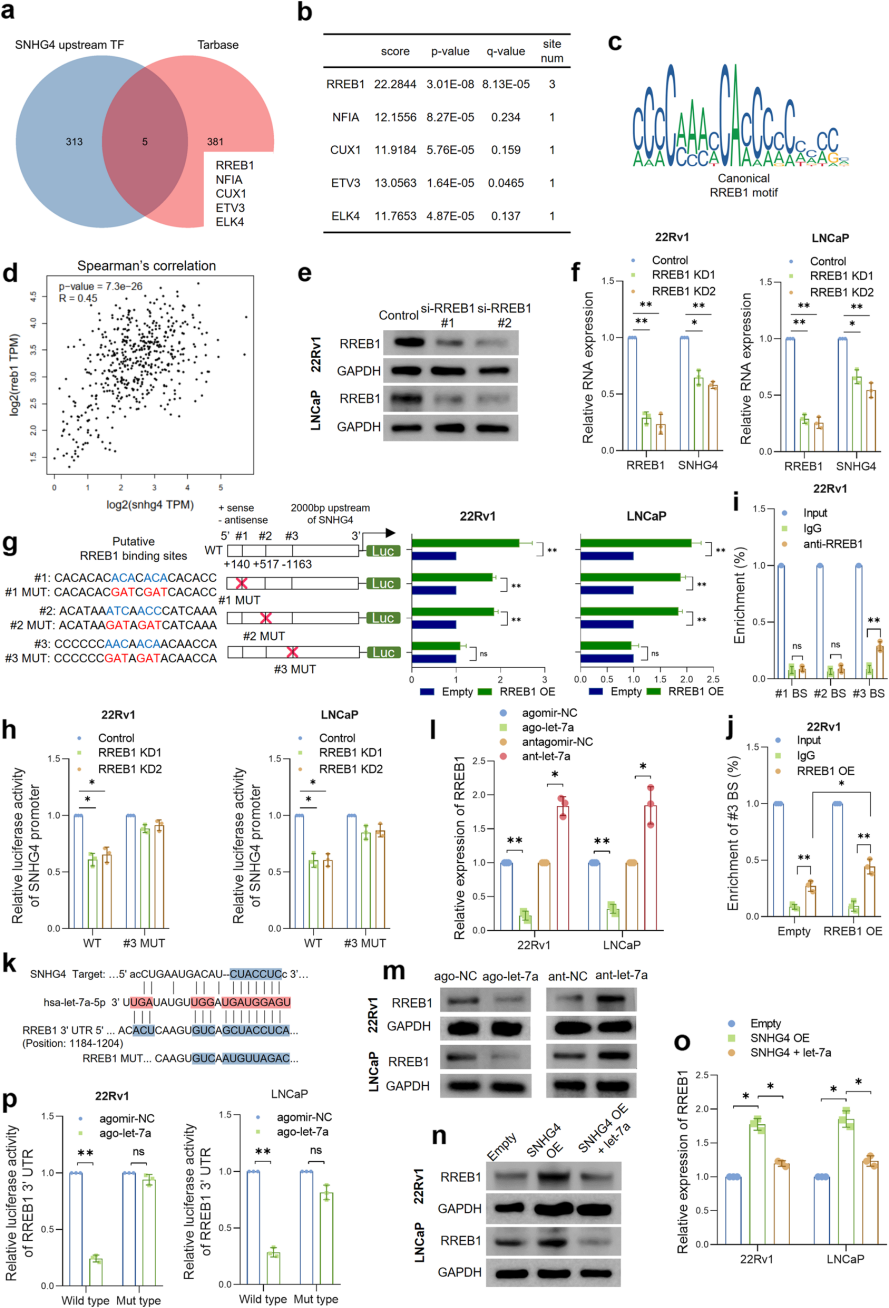
RREB1 regulates the transcription of SNHG4 in PCa cells, and SNHG4 modulates RREB1 expression through a SNHG4/let-7a-5p/RREB1 positive feedback loop. **a** Venn diagram showing that there were five genes across two gene sets (predicted SNHG4 upstream TFs and potential let-7 targeted mRNAs). **b** The table shows the prediction score, *p* value, *q*-value and number of binding sites of the five predicted SNHG4 upstream TFs. **c** The canonical motif of RREB1. **d** Spearman’s correlation coefficient test showed the correlation between the expression of SNHG4 and RREB1 in PCa tumor samples (*n* = 499, *R* = 0.45, *p* < 0.001). Western blot (**e**) and qRT‒PCR analysis (**f**) showed that RREB1 expression was significantly knocked down by either pair of siRNAs against RREB1. **g** Construction of luciferase reporter plasmids containing the wild-type SNHG4 promoter or either of the three BS-mutated SNHG4 promoters is shown here. A dual-luciferase reporter assay showed that mutation of #3 BS significantly reduced the interactions between RREB1 and the SNHG4 promoter. **h** A dual-luciferase reporter assay was performed to assess the interactions between RREB1 and the SNHG4 promoter. si-RREB1 significantly inhibited the luciferase activity of luciferase plasmids containing the wild-type SNHG4 promoter but had no effect on the transcription of the #3 BS mutated SNHG4 promoter. **i** ChIP assay suggested that #3 BS was significantly enriched in the DNA products pulled down by anti-RREB1 antibody in 22Rv1 cells, whereas no significant enrichment was observed in #1 and #2 BS. **j** RREB1 was overexpressed in 22Rv1 cells, and ChIP assays showed that overexpression of RREB1 increased the enrichment of #3 BS in the pulldown product. **k** Predicted sequence of binding sites between SNHG4, let-7a and the 3’UTR of RREB1. **l** and **m** qRT‒PCR and western blot showed that let-7a mimics significantly depleted RREB1 expression; in contrast, let-7a inhibitor notably increased RREB1 expression. **n** and **o** qRT‒PCR and western blot showed that SNHG4 overexpression significantly increased RREB1 expression, whereas restoration of let-7a partially neutralized the RREB1 overexpression that was induced by SNHG4 overexpression alone. **p** Dual luciferase reporter assay shows that let-7a overexpression inhibited the transcription of luciferase reporter vectors harboring the 3’UTR of RREB1 in 22Rv1 and LNCaP cells. ns indicates not significant, * indicates *p* < 0.05, ** indicates *p* < 0.01

Next, we aimed to construct a regulatory loop that may constitutively activate the transcription of SNHG4 in prostate cancer. We used MEME suite software to predict the potential transcription factors (TFs) that may bind to the promoter region of the SNHG4 gene, and we also predicted the potential let-7a target genes using Tarbase software. Consequently, we found five TFs that may be regulated by SNHG4 through the let-7 miRNA-mediated ceRNA network and had the potential to regulate the transcription of the SNHG4 gene (Fig. 9a). Among the five TFs, RREB1 had the highest prediction score and had three potential binding sites within the SNHG4 gene promoter (Fig. 9b). Moreover, as the effector of RAS signaling, RREB1 was reported to contribute to EMT, proliferation and invasiveness in multiple human cancers. The canonical motif of RREB1 is shown in Fig. 9c. We also found that SNHG4 was positively correlated with the expression of RREB1 in TCGA_PRAD datasets (Fig. 9d). Therefore, we hypothesized that RREB1 transcriptionally regulates SNHG4 expression in prostate cancer.

We used two pairs of siRNAs to knockdown RREB1 gene expression in PCa cells (Fig. 9e), and the qRT‒PCR results suggested that RREB1 knockdown notably inhibited the expression of SNHG4 (Fig. 9f). To investigate the binding sites (BSs) within the SNHG4 promoter, we mutated the predicted BSs and constructed four types of luciferase reporter plasmids that harbored the wild type, #1 BS mutant type, #2 BS mutant type or #3 BS mutant type of the SNHG4 gene. The luciferase reporter plasmids were cotransfected with empty vectors or RREB1-overexpressing plasmids into PCa cells. The results from the dual luciferase reporter assay showed that the #3 BS mutant blocked the interactions between RREB1 and SNHG4 (Fig. 9g). Next, luciferase reporter plasmids containing the #3 BS mutant were cotransfected with scramble control or siRNA against RREB1 into the cells, and a dual luciferase reporter assay was performed to assess the interactions between RREB1 and the #3 BS of SNHG4. Not surprisingly, the results confirmed our hypothesis (Fig. 9h). ChIP assays were then performed, and the results suggested that the #3 BS was significantly enriched in the DNA products pulled down by the anti-RREB1 antibody (Fig. 9i). In addition, RREB1 overexpression further increased the enrichment level of the #3 BS, which suggested that the #3 BS was targeted by RREB1 (Fig. 9j).

**Fig. 10:**
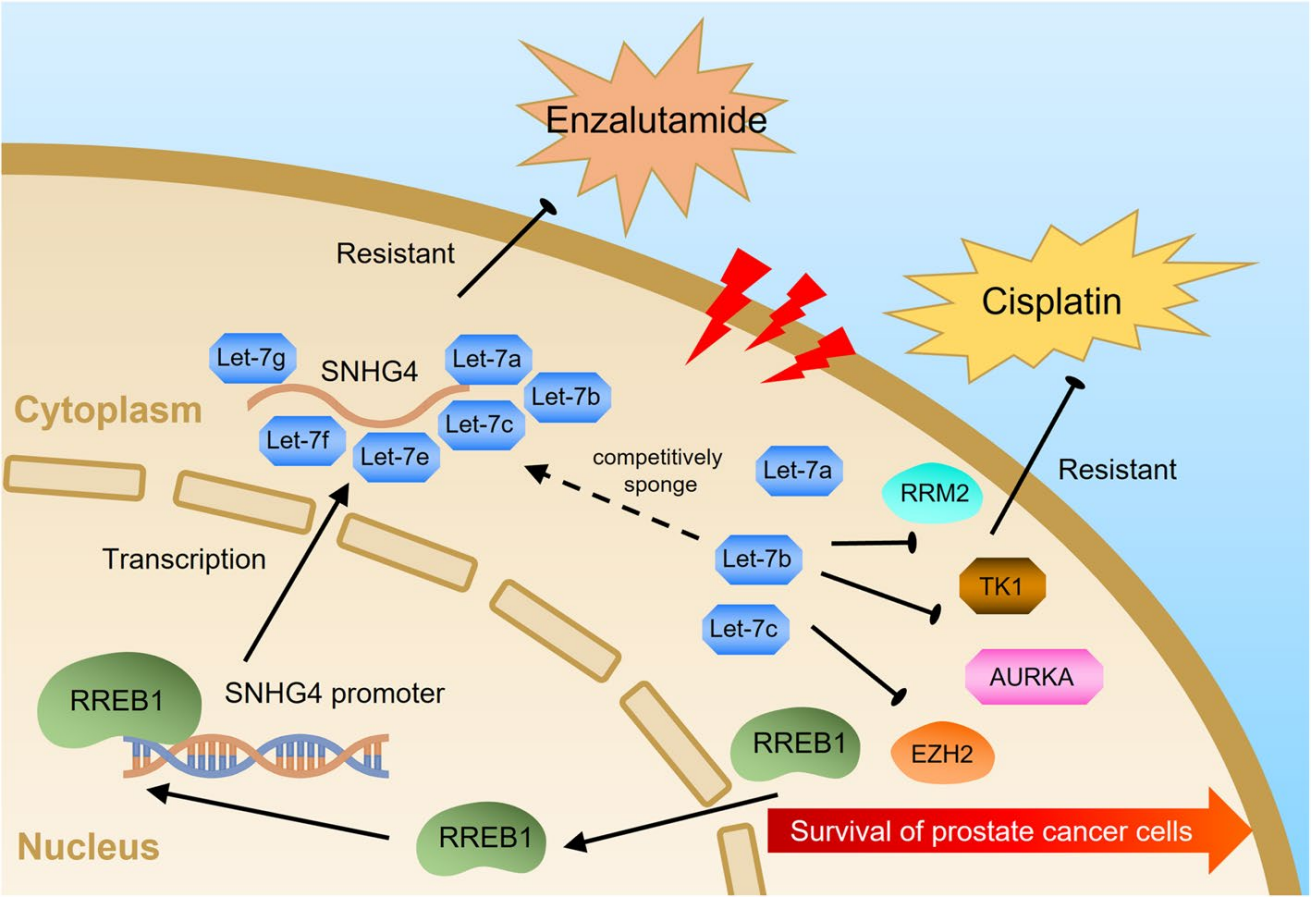
Proposed model of SNHG4 driving prostate cancer progression and enzalutamide resistance. SNHG4 regulates the cell cycle, cell senescence, DNA damage repair, cell proliferation and enzalutamide resistance by modulating RRM2, EZH2, AURKA and TK1 through a let-7 miRNA-mediated ceRNA regulatory network. In addition, RREB1 activates the transcription of SNHG4 and is regulated by the SNHG4/let-7/RREB1 feedback loop

We then examined whether SNHG4 regulated RREB1 through let-7a. The let-7a-5p response element within the 3’UTR of RREB1 is shown in Fig. 9k. qRT‒PCR and western blot analysis were performed in let-7a-overexpressing or let-7a-knockdown PCa cells, and the results suggested that RREB1 expression was significantly upregulated in response to let-7a knockdown, whereas let-7a overexpression dramatically decreased the expression level of RREB1 (Fig. 9l and m). We also noticed that SNHG4 overexpression was capable of upregulating RREB1 expression; in contrast, restoration of let-7a notably neutralized the RREB1 upregulation induced by SNHG4 overexpression (Fig. 9n and o). Finally, a dual luciferase reporter assay was performed to confirm that let-7a binds to the 3’UTR of the RREB1 gene (Fig. 9). Collectively, the above data suggested that RREB1 transcriptionally regulated SNHG4 gene expression and that SNHG4 was capable of regulating RREB1 through let-7a. The RREB1/SNHG4/let-7a regulatory loop may enhance the aggressiveness of prostate cancer (Fig. 10).

## Discussion

Prostate cancer has been drawing great attention from clinicians and biologists due to its increasing incidence, heterogeneity and therapeutic resistance [35]. With the development of omics techniques, accumulating evidence has identified novel lncRNAs in the initiation and progression of human cancers [36, 37]. For example, lncRNA PCAT1 reportedly suppresses the radioimmune response by regulating cGAS/STING signaling in non-small cell lung cancer [38]. PCAT1 activates AKT and NF-kB signaling in CRPC [39]. In melanoma, lncRNA LENOX interacts with RAP2C to regulate metabolism and promote resistance to MAPK inhibition [40]. SNHG4 is a well-known oncogene and participates in a variety of mechanisms to enhance tumor progression, such as in colorectal cancer [22] and lung cancer [21]. In prostate cancer, SNHG4 is reported to sponge miR-377 and regulate ZIC expression [24]. Nonetheless, the underlying mechanisms of SNHG4 in modulating the malignancy of PCa and affecting the therapeutic resistance of PCa remain unclear. In this study, for the first time, we uncovered the mechanisms by which SNHG4 facilitates the survival of PCa cells and resistance to enzalutamide, and we investigated a positive feedback loop that may enhance the overexpression of SNHG4 in prostate cancer.

We identified that RREB1 activates the transcription of SNHG4 in PCa cells. RREB1 is a well-known RAS transcription effector and mediates TGF-β-induced epithelial-to-mesenchymal transitions (EMTs) in human cancers [41]. Mutations in RREB1 have been observed in pancreatic ductal adenocarcinoma [42] and gastric cancer [42]. As a transcription factor, RREB1 was identified to upregulate lncRNA AGAP2-AS1 and promote the progression of pancreatic cancer [43]. In colorectal cancer, RREB1 binds to the AKT1 promoter and activates AKT transcription [44]. In glioma, RREB1 transcriptionally upregulates U2AF65 and improves the stability of circNCAPG [45]. Little is known about the functions of RREB1 in prostate cancer. Interestingly, it has been reported that RREB1 acts as an AR coregulator and binds to the PSA promoter [46]. In the present study, we have demonstrated that RREB1 activates the transcription of SNHG4, and the SNHG4/let-7a/RREB1 positive feedback loop promotes the overexpression of SNHG4 and RREB1 in PCa cells. However, the functions and mechanisms of RREB1 in regulating the tumorigenesis of prostate cancer need to be further investigated.

The let-7 (lethal-7) family of microRNAs, which consists of 10 mature miRNAs derived from 13 precursor genes, has been recognized to possess various biological functions, such as cell proliferation, differentiation and regulation of cancer stemness and development [47]. Let-7 is downregulated in prostate cancer and acts as a tumor suppressor, and overexpression of let-7 induces G2/M phase cell cycle arrest [48]. Moreover, let-7 has been found to maintain the cancer stem-like cell (CSC) phenotypes of prostate cancer, let-7 expression is controlled by LIN28A and LIN28B [49], and loss of let-7 increases the expression of SOX2 and promotes the cell transformation and expansion of prostate CSCs [50]. Let-7 is also reported to regulate EZH2 to modulate CSC signatures of prostate cancer [51]. In this study, we demonstrated that RRM2, EZH2, AURKA, TK1 and RREB1 are putative targets of let-7, and by sponging let-7, SNHG4 releases the expression of these genes and facilitates cell proliferation, senescence, cell cycle arrest, DNA damage repair and therapeutic resistance. Whether SNHG4/let-7 enhances the CSC phenotypes of prostate cancer remains to be further investigated.

RRM2 has been identified to be overexpressed in prostate cancer and significantly correlated with poor survival of PCa patients [52]. Mechanistically, RRM2 is a key enzyme for dNTP synthesis, and dNTP supplementation is essential for DNA synthesis and DNA repair. Loss of RRM2 has been reported to induce cell cycle arrest, cell senescence and SASP phenotypes, and the RRM2 inhibitor COH29 has been proven to exert notable antitumor effects in prostate cancer cells [53]. Furthermore, a bioinformatic study defining the RRM2 signature in PCa suggested that the RRM2 signature may predict enzalutamide resistance, and RRM2 overexpression suggests an immunosuppressive tumor microenvironment in prostate cancer [52], indicating that RRM2 may be implicated in the therapeutic resistance of ADT and immunotherapy. Another study provided evidence that knockdown of RRM2 enhances the antitumor efficiency of sunitinib and anti-PD-1 therapy in renal cancer [54], which further supported the potential role of RRM2 as a target to facilitate therapeutic resistance. In our study, we verified that RRM2 knockdown leads to cell cycle arrest, cell senescence and hampered DNA damage repair, and overexpression of SNHG4 causes upregulation of RRM2 and enhances enzalutamide resistance. Nonetheless, the biological functions of RRM2 in regulating cancer development and therapeutic resistance remain to be elucidated.

Indeed, there are some limitations in this study. First, at the beginning of the study, our analysis revealed RRM2 and NUSAP1 to be strong predictors of prostate cancer, and we believe that NUSAP1 is another interesting gene that is worth investigating. Second, although we have identified that RREB1 acts as an upstream transcription factor of SNHG4, the biological functions and mechanisms of RREB1 in the tumorigenesis of PCa and drug resistance remain unclear. Third, let-7 has been recognized to regulate the CSC phenotypes of human cancers, and since we have proven that SNHG4 sponges let-7, whether SNHG4 affects the stemness of prostate cancer is worthy of investigation. Finally, RRM2 has been found to be correlated with the malignant phenotypes of prostate cancer, and bioinformatic analysis suggested that RRM2 may have impacts on tumor microenvironment reconstruction and enzalutamide resistance. The hypothesis and mechanisms still need to be further studied.

## Conclusions

Our study revealed the prognostic value and biological functions of SNHG4 in stimulating prostate cancer progression. SNHG4 is highly expressed in PCa tissues and correlated with poor overall survival and clinical outcomes of PCa patients. Let-7 mediates a ceRNA network through which SNHG4 promotes the expression of RRM2, EZH2, AURKA and TK1 to promote DNA damage repair, the cell cycle, cell proliferation and enzalutamide resistance. RREB1 activates the transcription of SNHG4 in PCa cells, and the RREB1/SNHG4/let-7 positive feedback loop maintains the expression and carcinogenic functions of RREB1 and SNHG4. Our study uncovered a novel molecular mechanism of lncRNA SNHG4 in driving prostate cancer progression and enzalutamide resistance, revealing the critical roles and therapeutic potential of RREB1, SNHG4 and let-7 miRNAs in anticancer therapy.

### Supplementary Information

**Additional file 1: Figure S1. **Flow chart of the study.** Figure S2.** a. The staining intensity of RRM2 was significantly stronger in PCa tumors (*n*=20) than in adjacent normal prostate tissues (*n*=20) and BPH tissues (*n*=10) by IHC staining. b. qRT-PCR analysis suggested that RRM2 was highly expressed in PCa cell lines (DU145, PC3, 22Rv1 and LNCaP) compared to normal prostate epithelial cell line RWPE-1. c. RRM2 levels were significantly decreased or increased in response to RRM2 knockdown or overexpression in 22Rv1 and LNCaP cells by qRT‒PCR and western blotting. d. Knockdown of RRM2 notably induced cell cycle arrest in the G1 stage in 22Rv1 and LNCaP cells. The image for each experiment is shown in Fig. 2h. e. The correlation between the expression levels of NEAT1 and RRM2 in PCa tumor samples (*n*=499) was not significant. The data were obtained from the TCGA_PRAD dataset. f. Knockdown of NEAT1 had no effect on RRM2 levels in RV-a and LNCaP cells, as determined by western blotting. **g**. Let-7a-5p levels were significantly decreased or increased in response to transfection of let-7a-5p inhibitor or mimics in 22Rv1 and LNCaP cells by qRT‒PCR. h. High SNHG4 levels indicate poor progression free interval in PCa patients, data from the TCGA_PRAD dataset. i. qRT-PCR analysis suggested that SNHG4 was highly expressed in PCa cell lines (DU145, PC3, 22Rv1 and LNCaP) compared to normal prostate epithelial cell line RWPE-1. j. SNH4 coexpressed genes were enriched in the biological term “Cell Cycle”, indicating a potential role of SNHG4 in regulating the cell cycle of PCa cells. k. Representative ISH/IHC staining images of the indicated gene/protein expression in a series of clinical pathological sections from 30 PCa patients. The staining intensity of each gene/protein was scored as 0 to 5 (0: no staining, 1: very weak staining, 2: weak staining, 3: medium staining, 4: strong staining, 5: very strong staining), and 1-3 were classified as low expression, whereas 4-5 were defined as high expression. l. SNHG4 level is positively correlated with each indicated protein in PCa tumors (*p*<0.05, Fisher’s exact test).** Figure S3.** a. The knockdown efficiency of siRNAs against each indicated gene was measured by qRT‒PCR and western blotting. b. Knockdown of EZH2, AURKA or TK1 reduced the proliferation of PCa cells. Representative images of EdU staining of Fig. 6c and d. Magnification: 100X.** Figure S4.** a. Knockdown of each indicated gene significantly induced cell senescence in LNCaP cells, and senescent cell numbers were counted and compared. Magnification: 200X. b and c. qRT-PCR analysis showed that SNHG4 levels in 22Rv1 and LNCaP cells were significantly decreased in response to SNHG4 knockdown, whereas let-7a knockdown or RRM2 overexpression rescued SNHG4 expression. d. Western blot analysis showed that SNHG4 knockdown significantly decreased RRM2 expression, whereases let-7a knockdown or RRM2 overexpression rescued RRM2 expression in 22Rv1 and LNCaP cells. e. Knockdown of SNHG4 reduced the proliferation of PCa cells, whereas let-7a knockdown or RRM2 overexpression rescued cell proliferation of 22Rv1 and LNCaP cells. Representative images of EdU staining of Fig. 7b. Magnification: 100X.** Figure S5.** a. γ-H2AX foci were detected in PCa cells treated with negative control, SNHG4 knockdown, double knockdown of SNHG4 and let-7a, or SNHG4 knockdown with RRM2 overexpression by immunofluorescence staining. The indicated cells were treated with Docetaxel (10 nM) for 24 hours. Magnification: 200X. b. SNHG4 knockdown significantly induced cell cycle arrest in G1 phase, whereas knockdown of let-7a or RRM2 overexpression rescued the arrested cell cycle. The cell cycle was measured by FACS in pretreated 22Rv1 and LNCaP cells. **Supplemental Methods.**

**Additional file 2: Table S1.** Associations between expression of SNHG4/RRM2 and clinicopathological characteristics of 60 PCa patients. 

**Additional file 3: Table S2.** Clinical information of prostate cancer patients included in this study.

**Additional file 4: Table S3.** Primer sequences for qRT-PCR.

## Data Availability

The dataset supporting the conclusions of this article is available from the corresponding author upon reasonable request. The datasets generated during and analyzed during the current study are available in the TCGA and GEO repository, TCGA: https://portal.gdc.cancer.gov/, GEO: https://www.ncbi.nlm.nih.gov/geo/).

## Abbreviations

SNHG4: Small nucleolar RNA host gene 4
RRM2: Ribonucleotide reductase regulatory subunit M2
NUSAP1: Nucleolar and spindle associated protein 1
EZH2: Enhancer of zeste 2 polycomb repressive complex 2 subunit
TK1: Thymidine kinase 1
AURKA: Aurora kinase A
RREB1: Ras responsive element binding protein 1
