# Supplementary material for: LncRNA SNHG4 promotes prostate cancer cell survival and resistance to enzalutamide through a let-7a/RREB1 positive feedback loop and a ceRNA network
Source: J Exp Clin Cancer Res. 2023 Aug 18;42:209. doi: 10.1186/s13046-023-02774-2 (PMC10436424; doi:10.1186/s13046-023-02774-2)
Supplement: Supplementary file 1 — Additional file 1: Figure S1. Flow chart of the study. Figure S2. a. The staining intensity of RRM2 was significantly stronger in PCa tumors (n=20) than in adjacent normal prostate tissues (n=20) and BPH tissues (n=10) by IHC staining. b. qRT-PCR analysis suggested that RRM2 was highly expressed in PCa cell lines (DU145, PC3, 22Rv1 and LNCaP) compared to normal prostate epithelial cell line RWPE-1. c. RRM2 levels were significantly decreased or increased in response to RRM2 knockdown or overexpression in 22Rv1 and LNCaP cells by qRT‒PCR and western blotting. d. Knockdown of RRM2 notably induced cell cycle arrest in the G1 stage in 22Rv1 and LNCaP cells. The image for each experiment is shown in Fig. 2h. e. The correlation between the expression levels of NEAT1 and RRM2 in PCa tumor samples (n=499) was not significant. The data were obtained from the TCGA_PRAD dataset. f. Knockdown of NEAT1 had no effect on RRM2 levels in RV-a and LNCaP cells, as determined by western blotting. g. Let-7a-5p levels were significantly decreased or increased in response to transfection of let-7a-5p inhibitor or mimics in 22Rv1 and LNCaP cells by qRT‒PCR. h. High SNHG4 levels indicate poor progression free interval in PCa patients, data from the TCGA_PRAD dataset. i. qRT-PCR analysis suggested that SNHG4 was highly expressed in PCa cell lines (DU145, PC3, 22Rv1 and LNCaP) compared to normal prostate epithelial cell line RWPE-1. j. SNH4 coexpressed genes were enriched in the biological term “Cell Cycle”, indicating a potential role of SNHG4 in regulating the cell cycle of PCa cells. k. Representative ISH/IHC staining images of the indicated gene/protein expression in a series of clinical pathological sections from 30 PCa patients. The staining intensity of each gene/protein was scored as 0 to 5 (0: no staining, 1: very weak staining, 2: weak staining, 3: medium staining, 4: strong staining, 5: very strong staining), and 1-3 were classified as low expression, whereas 4-5 were defined as hi [file 13046_2023_2774_MOESM1_ESM.zip › Supplemental materials.docx]

**Supplemental Figure Legends**

**Figure S1**

Flow chart of the study.

**Figure S2**

**a**. The staining intensity of RRM2 was significantly stronger in PCa tumors (n=20) than in adjacent normal prostate tissues (n=20) and BPH tissues (n=10) by IHC staining. b. qRT-PCR analysis suggested that RRM2 was highly expressed in PCa cell lines (DU145, PC3, 22Rv1 and LNCaP) compared to normal prostate epithelial cell line RWPE-1. c. RRM2 levels were significantly decreased or increased in response to RRM2 knockdown or overexpression in 22Rv1 and LNCaP cells by qRT‒PCR and western blotting. **d**. Knockdown of RRM2 notably induced cell cycle arrest in the G1 stage in 22Rv1 and LNCaP cells. The image for each experiment is shown in Figure 2h. **e**. The correlation between the expression levels of NEAT1 and RRM2 in PCa tumor samples (n=499) was not significant. The data were obtained from the TCGA_PRAD dataset. **f**. Knockdown of NEAT1 had no effect on RRM2 levels in RV-a and LNCaP cells, as determined by western blotting. **g**. Let-7a-5p levels were significantly decreased or increased in response to transfection of let-7a-5p inhibitor or mimics in 22Rv1 and LNCaP cells by qRT‒PCR. **h**. High SNHG4 levels indicate poor progression free interval in PCa patients, data from the TCGA_PRAD dataset. i. qRT-PCR analysis suggested that SNHG4 was highly expressed in PCa cell lines (DU145, PC3, 22Rv1 and LNCaP) compared to normal prostate epithelial cell line RWPE-1. **j**. SNH4 coexpressed genes were enriched in the biological term “Cell Cycle”, indicating a potential role of SNHG4 in regulating the cell cycle of PCa cells. **k**. Representative ISH/IHC staining images of the indicated gene/protein expression in a series of clinical pathological sections from 30 PCa patients. The staining intensity of each gene/protein was scored as 0 to 5 (0: no staining, 1: very weak staining, 2: weak staining, 3: medium staining, 4: strong staining, 5: very strong staining), and 1-3 were classified as low expression, whereas 4-5 were defined as high expression. **l**. SNHG4 level is positively correlated with each indicated protein in PCa tumors (p<0.05, Fisher’s exact test).

**Figure S3**

**a**. The knockdown efficiency of siRNAs against each indicated gene was measured by qRT‒PCR and western blotting. **b**. Knockdown of EZH2, AURKA or TK1 reduced the proliferation of PCa cells. Representative images of EdU staining of Figure 6c and 6d. Magnification: 100X.

**Figure S4**

**a**. Knockdown of each indicated gene significantly induced cell senescence in LNCaP cells, and senescent cell numbers were counted and compared. Magnification: 200X. **b and c**. qRT-PCR analysis showed that SNHG4 levels in 22Rv1 and LNCaP cells were significantly decreased in response to SNHG4 knockdown, whereas let-7a knockdown or RRM2 overexpression rescued SNHG4 expression. **d**. Western blot analysis showed that SNHG4 knockdown significantly decreased RRM2 expression, whereases let-7a knockdown or RRM2 overexpression rescued RRM2 expression in 22Rv1 and LNCaP cells. **e**. Knockdown of SNHG4 reduced the proliferation of PCa cells, whereas let-7a knockdown or RRM2 overexpression rescued cell proliferation of 22Rv1 and LNCaP cells. Representative images of EdU staining of Figure 7b. Magnification: 100X.

**Figure S5**

**a**. γ-H2AX foci were detected in PCa cells treated with negative control, SNHG4 knockdown, double knockdown of SNHG4 and let-7a, or SNHG4 knockdown with RRM2 overexpression by immunofluorescence staining. The indicated cells were treated with Docetaxel (10 nM) for 24 hours. Magnification: 200X. **b**. SNHG4 knockdown significantly induced cell cycle arrest in G1 phase, whereas knockdown of let-7a or RRM2 overexpression rescued the arrested cell cycle. The cell cycle was measured by FACS in pretreated 22Rv1 and LNCaP cells.

**Supplemental Methods**

**Plasmids, cloning, mutagenesis and transfections**

RRM2 and RREB1 in the pcDNA3.1 vectors were purchased from GeneChem (Shanghai, China). The SNHG4 point mutation was generated by the Q5 Site-Directed Mutagenesis Kit (New England Biolabs, Ipswitch, MA). The full length of SNHG4 (NCBI Reference Sequence: NR_003141.4) or the binding site (CUACCUC) mutated type of SNHG4 was subcloned into the pLenti CMV GFP Puro (658-5) vectors. The siRNAs of SNHG4, RRM2, RREB1, AURKA, EZH2 or TK1 were purchased from GeneChem (Shanghai, China). The miRNA agomir and antagomir were synergized by and purchased from GenePharma (Shanghai, China).

Transfections were performed using Lipofectamine 3000 Reagent (Invitrogen) following the manufacturer’s protocol. Final concentrations for miRNA agomir or plasmids were 50 nM and 0,75 μg/ml. Cells were cultured in a six-well plate with 2 ml culture medium. The concentration for lentivirus transduction was 5 × 10^6^ transducing units of lentivirus. Stable cell lines were constructed using puromycin (200 μg ml^−1^).

**Gene Ontology (GO) Term and Kyoto Encyclopedia of Genes and Genomes (KEGG) Pathway Enrichment Analysis and Gene Set Enrichment Analysis (GSEA)**

Gene ontology (GO, http://geneontology.org/) is a widely used tool for annotating genes with functions, especially molecular functions (MF), biological pathways (BP), and cellular components (CC). KEGG is a collection of databases dealing with genomes, biological pathways, diseases, drugs, and chemical substances (www.kegg.jp/kegg/kegg1.html). GSEA is a computational method that allows the determination of classes of genes or proteins that are overrepresented in a large set of genes or proteins and may have a statistically significant association with disease phenotypes. The predefined gene set is from the MSigDB database (https://www.gsea-msigdb.org/gsea/msigdb/index.jsp). The enriched pathways were determined based on the nominal P value and the normalized enrichment score (NES).

**Kaplan‒Meier analysis**

The Kaplan‒Meier (KM) method was used to analyze the correlation between the expression of SNHG4, RRM2 or NUSAP1 and the survival of patients. The prognostic value of SNHG4, RRM2 and NUSAP1 in prostate cancer was assessed according to overall survival (OS) and progression-free interval (PFI) using Kaplan–Meier plotter. Kaplan‒Meier analysis was conducted based on RNA sequence datasets as well as clinical survival data of prostate cancer patients from TCGA. The KM analysis and KM curve were calculated by R software 3.6.3, the survminer package and the survival package.

**Immunohistochemistry (IHC) staining**

The expression of the indicated genes in tissue specimens was detected using an UltraSensitiveTM SP (Mouse/Rabbit) IHC kit (Maxin-Bio, Fuzhou, Fujian, China) according to the manufacturer’s instructions. Briefly, sections were first dewaxed in xylene and ethanol, and antigen retrieval was performed using a microwave for 10 min at 100°C. The sections were then incubated with antibodies for 1 h, followed by biotinylated anti-IgG antibody and streptavidin-biotinylated-complex horseradish peroxidase. DAB and hematoxylin were used for nuclear staining. The images were then captured by upright metallurgical microscope (Olympus, Tokyo, Japan) under an original magnification of 200x. Scoring was performed by two pathologists who counted the intensity of positive cells in a defined area. The staining intensity of each gene/protein was scored as 0 to 5 (0: no staining, 1: very weak staining, 2: weak staining, 3: medium staining, 4: strong staining, 5: very strong staining), and 1-3 were classified as low expression, whereas 4-5 were defined as high expression.

**In situ hybridization (ISH)**

ISH was performed using an ISH Kit (GenePharma, Shanghai, China) according to the manufacturer’s instructions. Briefly, formalin-fixed paraffin-embedded tissue slides were deparaffinized, deproteinated and prehybridized in prehybridization solution for 2 h at 42°C. Then, the sections were incubated in SNHG4-probe solution overnight at 42°C. The slides were then exposed to a streptavidin-peroxidase reaction system and stained with DAB and hematoxylin. The images were captured by upright metallurgical microscope (Olympus, Tokyo, Japan) under an original magnification of 200x. To assess the expression of SNHG4 in tissue sections, scoring was performed by two pathologists who counted the intensity of positive cells in a defined area. Staining intensity was scored as 0 to 5 (0: no staining, 1: very weak staining, 2: weak staining, 3: medium staining, 4: strong staining, 5: very strong staining), and 1-3 were classified as SNHG4-low expression, whereas 4-5 were defined as SNHG4-high expression.

**Western blotting assay**

Cells were harvested in RIPA lysis buffer (Beyotime, Beijing, China) and boiled for 10 min at 90°C. Fifty micrograms of protein extract from cultured cells were separated by 10% SDS-polyacrylamide gel electrophoresis (SDS‒PAGE), and the gels were subsequently electro-transferred onto polyvinylidene difluoride (PVDF) membranes (Millipore), followed by incubation with the indicated primary antibodies in 5% nonfat milk in TBS-T overnight at 4°C. On the next day, the membranes were washed for 15 min each and immediately incubated with anti-rabbit or anti-mouse horseradish peroxidase-conjugated secondary antibodies for 1 h at 37°C. The immunobands were visualized using ECL reagents (Transgen Biotechnology, Beijing, China) on a MicroChemi Chemiluminescent Imaging System (DNR Bio-Imaging Systems, Mahale HaHamisha, Jerusalem, Israel). The densitometric values were calculated by ImageJ 1.46r software (Wayne Rasband, National Institutes of Health, Bethesda, MA, USA), and the ratios of target protein to GAPDH were used to conduct the statistical analysis.

**RNA extraction and qRT‒PCR analysis**

Total RNA was extracted from cultured cell lines using TRIzol reagent (Invitrogen) and reverse transcribed with random primers using PrimeScript™ RT Master Mix (Takara) according to the manufacturer’s instructions. For microRNA detection, cDNA synthesis and quantitative real-time PCR were performed using a mercury LNA™ Universal RT microRNA PCR kit (Exiqon). qRT‒PCR was performed using SYBR® Premix Ex Taq™ (Takara) and a LightCyclerTM 480 II system (Roche). β-actin and U6 snRNA were employed as endogenous controls for mRNA/lncRNA and miRNA, respectively. The primers used to amplify the target genes were listed in Table S3.

The relative levels of gene expression were quantified and analyzed using LightCyclerTM 480 software 1.5.1.6.2 (Roche). The real-time value for each sample was averaged and compared using the 2-ΔΔCt method. Three independent experiments were performed to analyze the relative gene expression.

**Cell cycle by flow cytometry**

Cells (3 × 10^4^ per well) were seeded into 24-well culture plates, cultured for 24 h and then subjected to the indicated treatment. The pretreated cells were harvested, washed in ice-cold PBS and then fixed in ice-cold 75% ethanol in PBS. PI/RNase staining buffer (BD, San Diego, CA, USA) was added, and the cells were incubated at 4°C for 30 min. The results were measured by flow cytometry (Becton Dickinson Biosciences, San Jose, CA), and the data were analyzed using the ModFit LT software package.

**Luciferase reporter assay**

Luciferase reporters were generated based on the psiCHECK2 vector (Promega) and a Dual Luciferase Reporter Assay Kit (Promega) according to the manufacturer’s protocol. To assess the interactions between SNHG4 and let-7a, psiCHECK-SNHG4 wild type (5456 bp, chr5:139,274,131-139,279,586) or psiCHECK-SNHG4 mutant type were PCR amplified and cloned into the psiCHECK2 vector, and the luciferase reporter construct was cotransfected with agomir of let-7a-5p into the cells by Lipofectamine 3000 (Invitrogen) according to the manufacturer’s guidelines. To assess the interactions between target genes and let-7a, the wild-type or mutant 3’UTR of the indicated target gene was cloned into the psiCHECHk2 vector, and the luciferase reporter was relatively cotransfected with the negative control or agomir of let-7a-5p. The relative luciferase activity was measured by a Synergy HTX multimode microplate reader (BioTek).

**RNA immunoprecipitation assay.**

Pretreated 22Rv1 cells were lysed with TRIzol reagent (Invitrogen), and total RNA was extracted and prepared. One microgram of each RNA sample was incubated with 5 μg of anti-IgG or anti-AGO2 antibody overnight at 4°C, and the complexes were isolated with magnetic beads (Invitrogen). The genes present in the pull-down products were measured by real-time PCR.

**Chromatin immunoprecipitation assay.**

The chromatin immunoprecipitation (ChIP) assay was performed using a SimpleChiP™ Enzymatic Chromatin IP kit (Cell Signaling Technology, Danvers, MA, USA) according to the manufacturer’s protocol. Cells (4 × 10^7^) in five 150-mm culture dishes were treated with 1% formaldehyde to crosslink proteins to DNA and were collected. The chromatin was digested by micrococcal nuclease to a length of approximately 150-900 bp. The cross-linked chromatin was separately incubated with 10 μL of anti-RREB1 p50 antibody (Cell Signaling Technology), 3 μL of anti-IgG antibody (negative control, Cell Signaling Technology), or 3 μL of anti-histone H3 antibody (positive control, Cell Signaling Technology) overnight at 4°C with rotation. Protein G agarose beads were used to harvest the immunoprecipitant. After reverse crosslinking of protein/DNA complexes to free the DNA, qRT‒PCR was performed to assess the enrichment of target sequences. RPL30 (provided by the kit) was used as an internal reference. Primers used to amplify the indicated fragments were listed in Table S3. Precipitated DNA was also amplified for 25 cycles and resolved on a 1% agarose gel to evaluate the amplification of target DNA.

**Senescence-associated β-Gal assay**

Pretreated cells cultured in a 6-well plate were washed three times, 1 ml of β-galactosidase staining solution was added to each well, and the cells were fixed for 15 min at room temperature. Then, the solution was removed, the cells were washed with PBS three times, 1 ml of staining solution was added to each well, and the cells were incubated overnight at 37°C. The next day, the cells were washed with PBS three times, and microscopy images were then captured by upright metallurgical microscopy (Olympus, Tokyo, Japan) under an original magnification of 200x.

**γ-H2AX staining assay**

The DNA damage of PCa cells was measured by a DNA Damage Assay Kit by γ-H2AX Immunofluorescence (Beyotime). Pretreated cells were washed with PBS, fixed and washed three times, and 1 ml of immunostaining blocking solution was added to the cells at room temperature for 10-20 min. Then, the immunostaining blocking solution was removed, and the cells were incubated with γ-H2AX rabbit monoclonal antibody (1 ml/well, 50 μl of antibody) for 1 h and then incubated with 1 ml/well of anti-rabbit 488 antibody for 1 h. The cells were washed three times and stained with 1 ml/well of DAPI for 5 min. The localized green fluorescence of the γ-H2AX-positive cells was visualized using an inverted fluorescence microscope (Olympus, Tokyo, Japan) and captured under an original magnification of 200×. The γ-H2AX-positive cells were counted using ImageJ.

**Proliferation assay**

The capacity for cellular proliferation was measured using a Cell Counting Kit-8 (CCK-8) (Dojindo, Tokyo, Japan) and a cell colony formation assay according to the manufacturer’s protocol. The absorbance value was measured at 450 nm to determine cell viability using a 96-well plate reader. For the cell colony formation assay, the cells were plated in 24-well plates (300 cells per well) and incubated for 14 days in complete medium. Colonies were fixed with 10% formaldehyde for 10 min and stained with 1.0% crystal violet for 5 min. The number of colonies, defined as > 50 cells/colony, was counted. 5-Ethynyl-2’- deoxyuridine (EdU) staining was performed using a BeyoClick™ EdU Cell Proliferation Kit with Alexa Fluor 488 (Beyotime). Cells were cultured in a 6-well plate and subjected to the indicated treatment. Then, 2X EdU buffer was added to the medium to a final concentration of 10 μM. After 24 hours, the cells were fixed and washed three times, and 0.5 ml of Click Additive Solution was added to each well. The plate was then gently shaken and incubated for 30 minutes away from light. The red fluorescence of the proliferative cells was visualized using an inverted fluorescence microscope (Olympus, Tokyo, Japan) and captured under an original magnification of 100×. The proliferative cells were counted using ImageJ.

**ATP assay**

ATP assays were performed using an ATP assay kit (Beyotime). The cells were cultured in a 6-well plate and subjected to the indicated treatment. Then, the medium was removed, and the cells were lysed by adding 200 μl of lysate buffer per well. Cells were centrifuged at 4°C and 12,000 × g for 5 min after lysis, and the supernatant was collected for subsequent assays. The ATP standard solution was diluted with ATP detection lysate to concentrations of 0.01, 0.03, 0.1, 0.3, 1, 3, and 10 μM to prepare the standard curve of ATP concentration. A 24-well plate was prepared, and 100 μl of ATP assay solution was added to each well at room temperature for 5 min. Then, 20 μl of sample or standard solution was added to each well, and after at least 2 seconds, the relative light unit (RLU) value was measured by a Synergy HTX multimode microplate reader (BioTek). The concentration of ATP in each sample was calculated from the standard curve.
