# Supplementary material for: LncRNA SNHG4 promotes prostate cancer cell survival and resistance to enzalutamide through a let-7a/RREB1 positive feedback loop and a ceRNA network
Source: J Exp Clin Cancer Res. 2023 Aug 18;42:209. doi: 10.1186/s13046-023-02774-2 (PMC10436424; doi:10.1186/s13046-023-02774-2)
Supplement: Supplementary file 1 — Additional file 1: Figure S1. Flow chart of the study. Figure S2. a. The staining intensity of RRM2 was significantly stronger in PCa tumors (n=20) than in adjacent normal prostate tissues (n=20) and BPH tissues (n=10) by IHC staining. b. qRT-PCR analysis suggested that RRM2 was highly expressed in PCa cell lines (DU145, PC3, 22Rv1 and LNCaP) compared to normal prostate epithelial cell line RWPE-1. c. RRM2 levels were significantly decreased or increased in response to RRM2 knockdown or overexpression in 22Rv1 and LNCaP cells by qRT‒PCR and western blotting. d. Knockdown of RRM2 notably induced cell cycle arrest in the G1 stage in 22Rv1 and LNCaP cells. The image for each experiment is shown in Fig. 2h. e. The correlation between the expression levels of NEAT1 and RRM2 in PCa tumor samples (n=499) was not significant. The data were obtained from the TCGA_PRAD dataset. f. Knockdown of NEAT1 had no effect on RRM2 levels in RV-a and LNCaP cells, as determined by western blotting. g. Let-7a-5p levels were significantly decreased or increased in response to transfection of let-7a-5p inhibitor or mimics in 22Rv1 and LNCaP cells by qRT‒PCR. h. High SNHG4 levels indicate poor progression free interval in PCa patients, data from the TCGA_PRAD dataset. i. qRT-PCR analysis suggested that SNHG4 was highly expressed in PCa cell lines (DU145, PC3, 22Rv1 and LNCaP) compared to normal prostate epithelial cell line RWPE-1. j. SNH4 coexpressed genes were enriched in the biological term “Cell Cycle”, indicating a potential role of SNHG4 in regulating the cell cycle of PCa cells. k. Representative ISH/IHC staining images of the indicated gene/protein expression in a series of clinical pathological sections from 30 PCa patients. The staining intensity of each gene/protein was scored as 0 to 5 (0: no staining, 1: very weak staining, 2: weak staining, 3: medium staining, 4: strong staining, 5: very strong staining), and 1-3 were classified as low expression, whereas 4-5 were defined as hi [file 13046_2023_2774_MOESM1_ESM.zip › FIgure S.pdf]

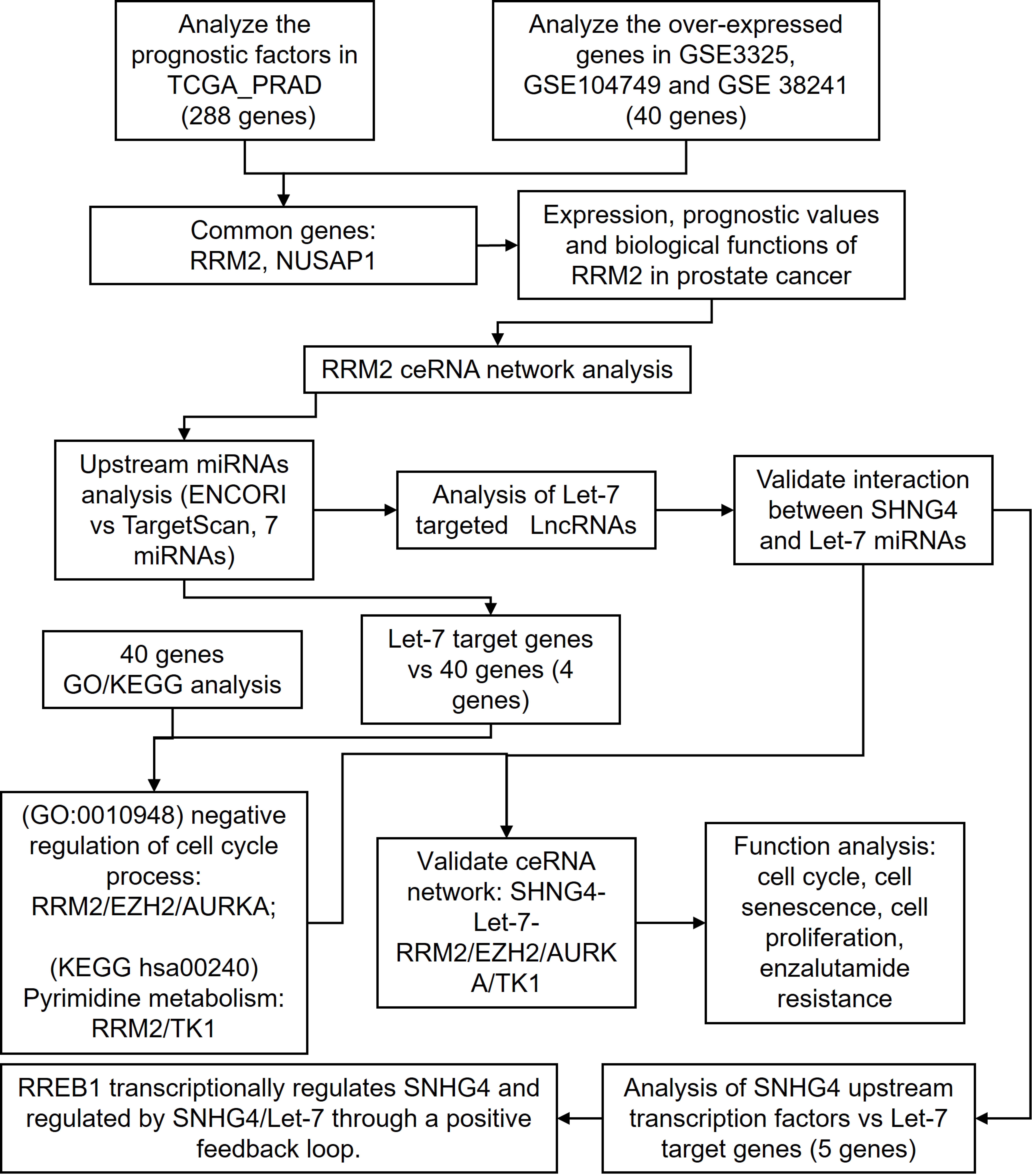

Analyze the prognostic factors in TCGA_PRAD (288 genes)
Analyze the over-expressed genes in GSE3325, GSE104749 and GSE 38241 (40 genes)
Common genes: RRM2, NUSAP1
Expression, prognostic values and biological functions of RRM2 in prostate cancer
RRM2 ceRNA network analysis
Upstream miRNAs analysis (ENCORI vs TargetScan, 7 miRNAs)
Analysis of Let-7 targeted LncRNAs
Validate interaction between SHNG4 and Let-7 miRNAs
40 genes GO/KEGG analysis
Let-7 target genes vs 40 genes (4 genes)
(GO:0010948) negative regulation of cell cycle process: RRM2/EZH2/AURKA;
(KEGG hsa00240) Pyrimidine metabolism: RRM2/TK1
Validate ceRNA network: SHNG4-Let-7-RRM2/EZH2/AURKA/TK1
Function analysis: cell cycle, cell senescence, cell proliferation, enzalutamide resistance
RREB1 transcriptionally regulates SNHG4 and regulated by SNHG4/Let-7 through a positive feedback loop.
Analysis of SNHG4 upstream transcription factors vs Let-7 target genes (5 genes)

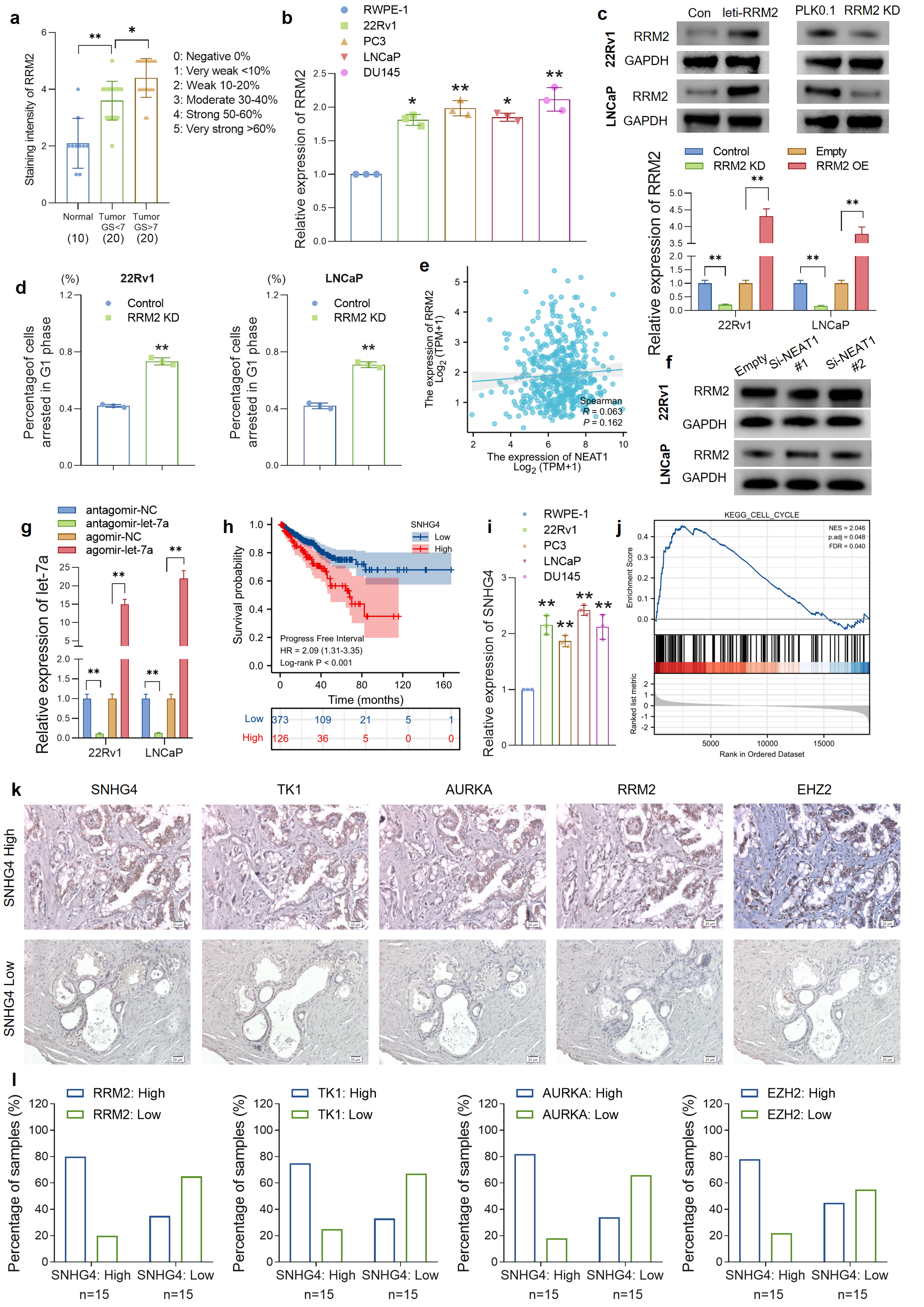

a
Staining intensity of RRM2
Normal (10)
Tumor GS<7 (20)
Tumor GS>7 (20)
0: Negative 0%
1: Very weak <10%
2: Weak 10-20%
3: Moderate 30-40%
4: Strong 50-60%
5: Very strong >60%
b
RWPE-1
22Rv1
PC3
LNCaP
DU145
Relative expression of RRM2
c
Con
leti-RRM2
PLK0.1
RRM2 KD
22Rv1
LNCaP
RRM2
GAPDH
Control
RRM2 KD
Empty
RRM2 OE
Relative expression of RRM2
d
22Rv1
LNCaP
(%)
Control
RRM2 KD
Percentageof cells arrested in G1 phase
e
The expression of RRM2 Log2 (TPM+1)
The expression of NEAT1 Log2 (TPM+1)
Spearman
R = 0.063
P = 0.162
f
Empty
Si-NEAT1 #1
Si-NEAT1 #2
g
antagomir-NC
antagomir-let-7a
agomir-NC
agomir-let-7a
Relative expression of let-7a
h
SNHG4
Low
High
Survival probability
Progress Free Interval
HR = 2.09 (1.31-3.35)
Log-rank P < 0.001
Time (months)
Low 373 109 21 5 1
High 126 36 5 0 0
i
Relative expression of SNHG4
j
KEGG_CELL_CYCLE
NES = 2.046
p.adj = 0.048
FDR = 0.040
Enrichment Score
Ranked list metric
Rank in Ordered Dataset
k
SNHG4
TK1
AURKA
RRM2
EHZ2
SNHG4 High
SNHG4 Low
l
RRM2: High
RRM2: Low
TK1: High
TK1: Low
AURKA: High
AURKA: Low
EZH2: High
EZH2: Low
Percentage of samples (%)
SNHG4: High n=15
SNHG4: Low n=15

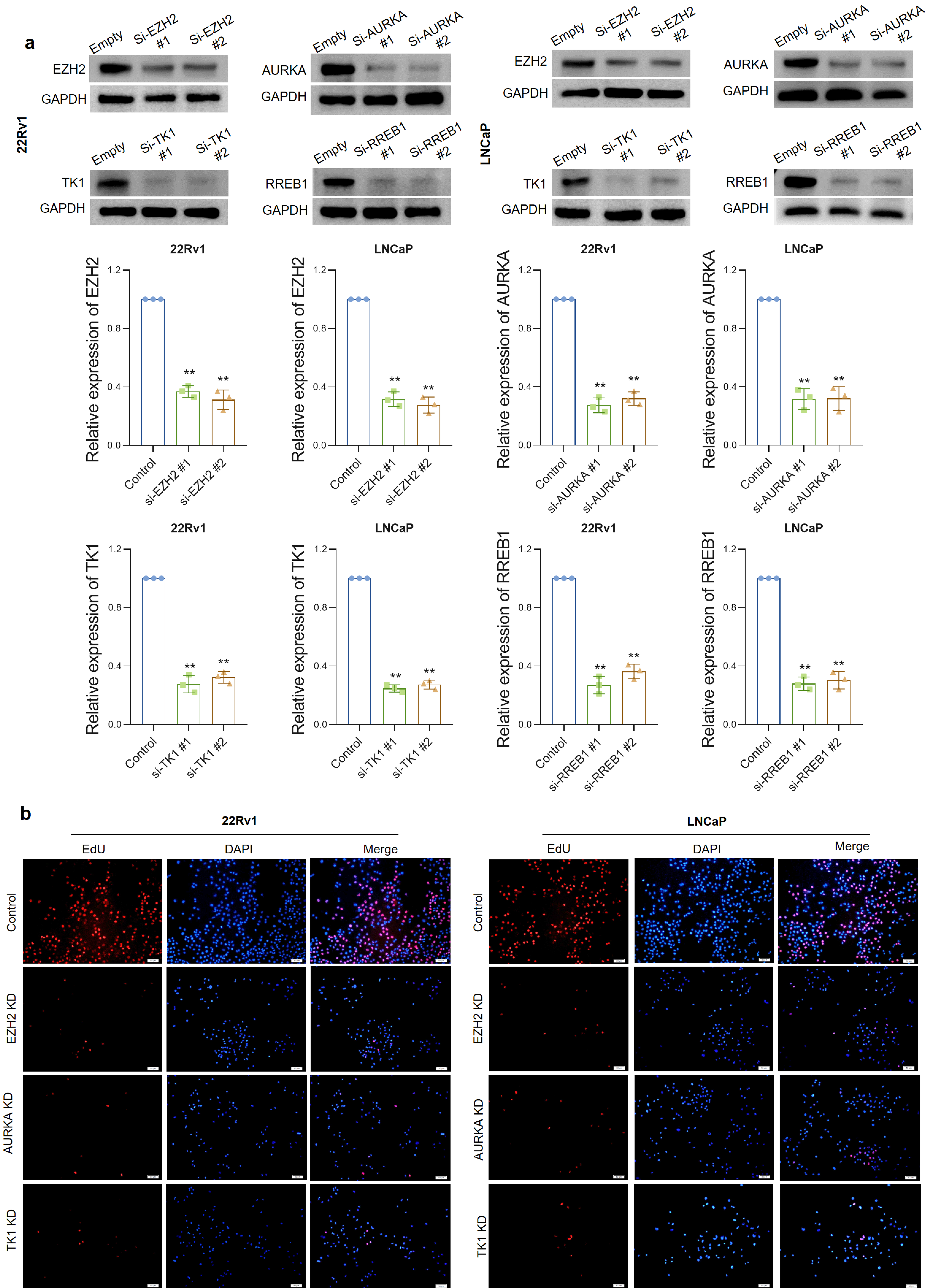
a
22Rv1
LNCaP
Empty
Si-EZH2 #1
Si-EZH2 #2
Si-AURKA #1
Si-AURKA #2
Si-TK1 #1
Si-TK1 #2
Si-RREB1 #1
Si-RREB1 #2
EZH2
AURKA
TK1
RREB1
GAPDH
Relative expression of EZH2
Relative expression of AURKA
Relative expression of TK1
Relative expression of RREB1
Control
si-EZH2 #1
si-EZH2 #2
si-AURKA #1
si-AURKA #2
si-TK1 #1
si-TK1 #2
si-RREB1 #1
si-RREB1 #2
b
EdU
DAPI
Merge
Control
EZH2 KD
AURKA KD
TK1 KD

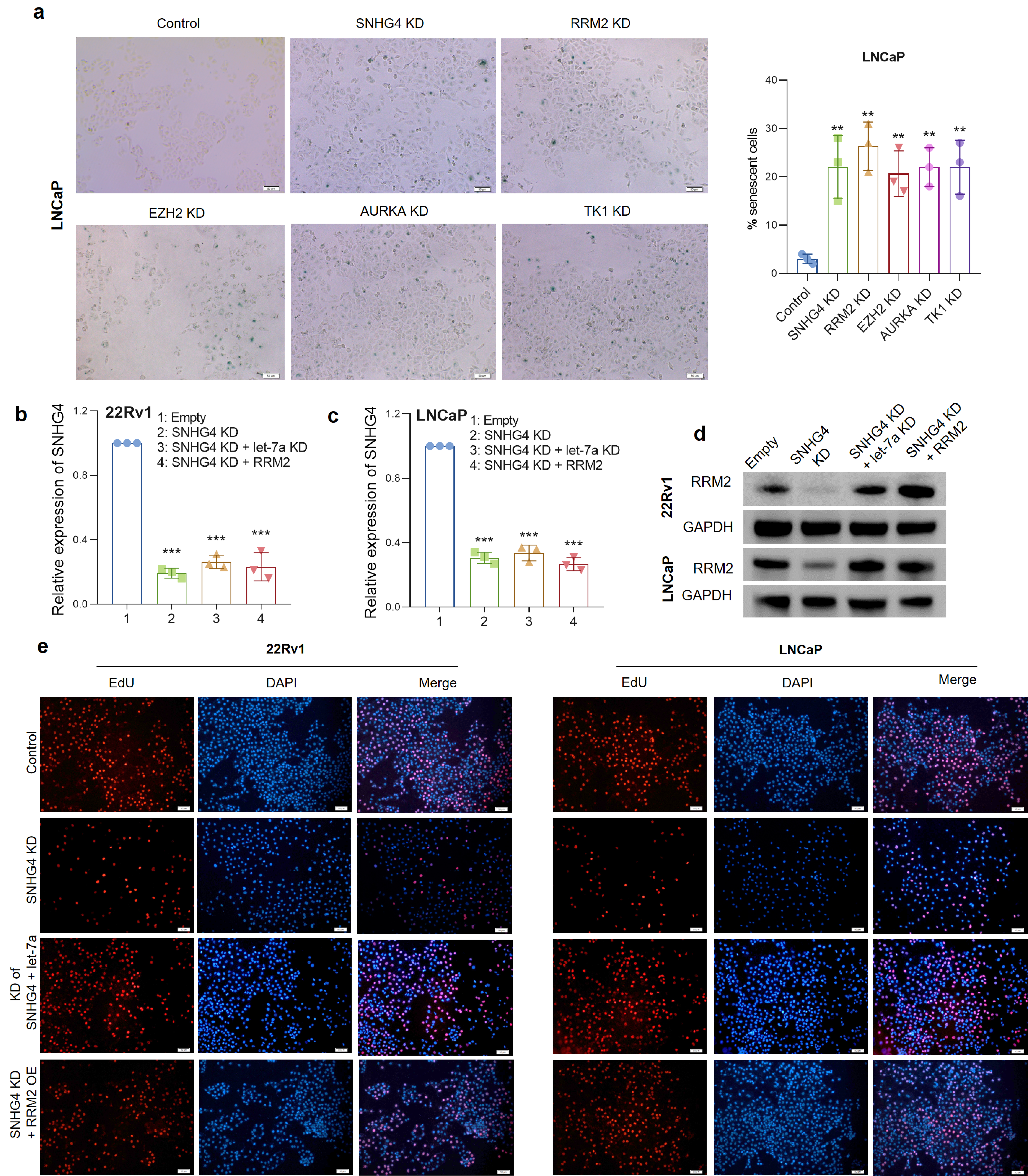
a
Control
SNHG4 KD
RRM2 KD
EZH2 KD
AURKA KD
TK1 KD
LNCaP
LNCaP
% senescent cells
0
10
20
30
40
**
Control
SNHG4 KD
RRM2 KD
EZH2 KD
AURKA KD
TK1 KD
b
22Rv1
1: Empty
2: SNHG4 KD
3: SNHG4 KD + let-7a KD
4: SNHG4 KD + RRM2
Relative expression of SNHG4
0.0
0.4
0.8
1.2
***
1
2
3
4
c
LNCaP
1: Empty
2: SNHG4 KD
3: SNHG4 KD + let-7a KD
4: SNHG4 KD + RRM2
Relative expression of SNHG4
d
Empty
SNHG4 KD
SNHG4 KD + let-7a KD
SNHG4 KD + RRM2
22Rv1
RRM2
GAPDH
LNCaP
RRM2
GAPDH
e
22Rv1
LNCaP
EdU
DAPI
Merge
Control
SNHG4 KD
KD of SNHG4 + let-7a
SNHG4 KD + RRM2 OE

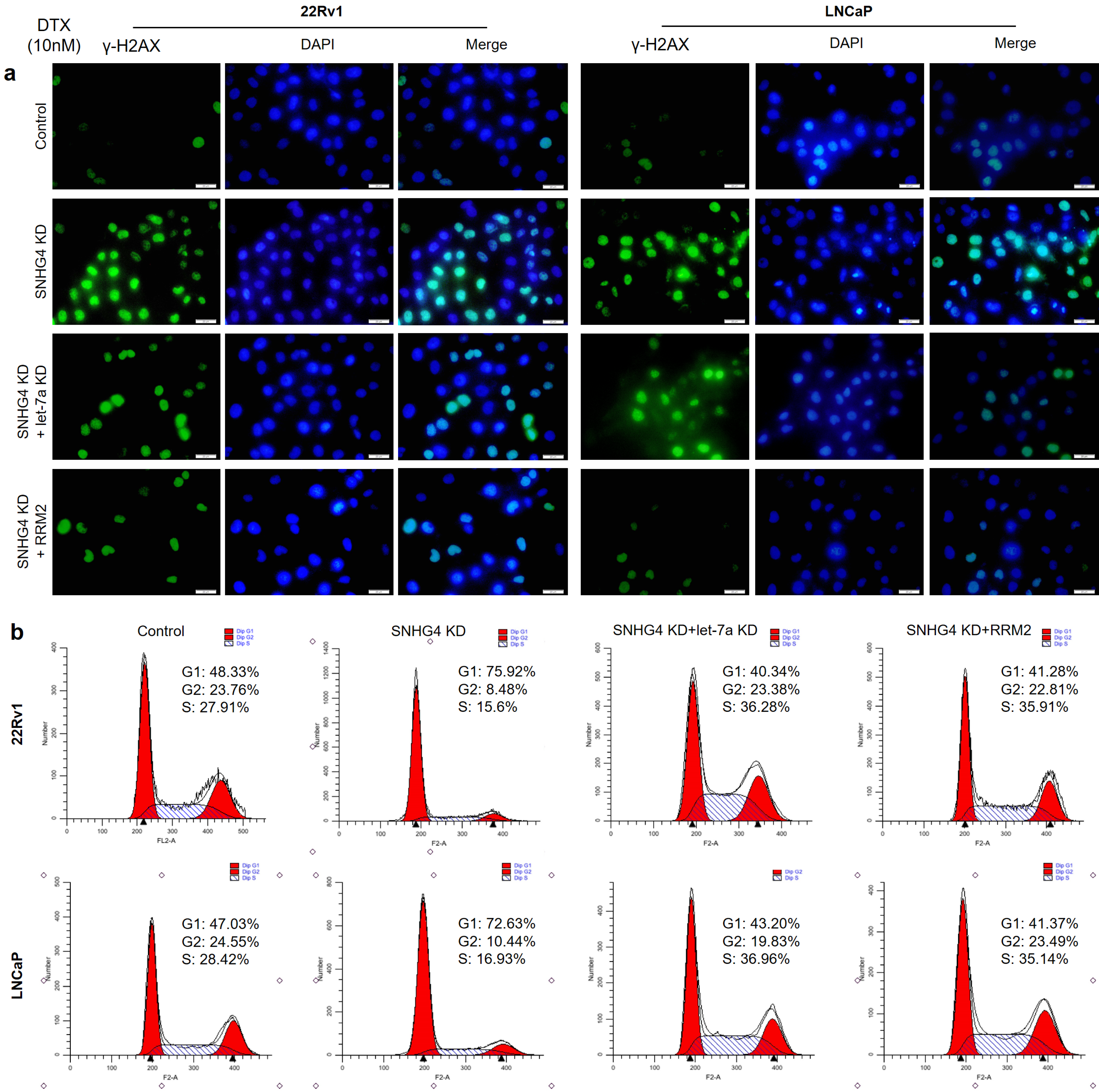

DTX (10nM)
22Rv1
LNCaP
γ-H2AX
DAPI
Merge
a
Control
SNHG4 KD
SNHG4 KD + let-7a KD
SNHG4 KD + RRM2
b
Control
SNHG4 KD
SNHG4 KD+let-7a KD
SNHG4 KD+RRM2
G1: 48.33%
G2: 23.76%
S: 27.91%
G1: 75.92%
G2: 8.48%
S: 15.6%
G1: 40.34%
G2: 23.38%
S: 36.28%
G1: 41.28%
G2: 22.81%
S: 35.91%
G1: 47.03%
G2: 24.55%
S: 28.42%
G1: 72.63%
G2: 10.44%
S: 16.93%
G1: 43.20%
G2: 19.83%
S: 36.96%
G1: 41.37%
G2: 23.49%
S: 35.14%
