# Supplementary material for: LncRNA SNHG4 promotes prostate cancer cell survival and resistance to enzalutamide through a let-7a/RREB1 positive feedback loop and a ceRNA network
Source: J Exp Clin Cancer Res. 2023 Aug 18;42:209. doi: 10.1186/s13046-023-02774-2 (PMC10436424; doi:10.1186/s13046-023-02774-2)
Supplement: Supplementary file 4 — Additional file 4: Table S3. Primer sequences for qRT-PCR. [file 13046_2023_2774_MOESM4_ESM.docx]

**Table S3: Primer sequences for qRT-PCR**

| **Name** | **Primer** | **Sequence (5'-3')** |
| --- | --- | --- |
| SNHG4 | Forward | AGTAGGGCATCCTTCACCC |
|  | Reverse | TGGGGTCCTGATGGAGGTAG |
| RRM2 | Forward | CACGGAGCCGAAAACTAAAGC |
|  | Reverse | TCTGCCTTCTTATACATCTGCCA |
| EZH2 | Forward | AATCAGAGTACATGCGACTGAGA |
|  | Reverse | GCTGTATCCTTCGCTGTTTCC |
| AURKA | Forward | GAGGTCCAAAACGTGTTCTCG |
|  | Reverse | ACAGGATGAGGTACACTGGTTG |
| TK1 | Forward | GGGCAGATCCAGGTGATTCTC |
|  | Reverse | TGTAGCGAGTGTCTTTGGCATA |
| RREB1 | Forward | AGGTTCAGACCTATCTTCCATCA |
|  | Reverse | CTGCCAATCCGATTTGGTCCT |
| IL1-a | Forward | AGTAGCAACCAACGGGAAGG |
|  | Reverse | AAGGTGCTGACCTAGGCTTG |
| IL1-b | Forward | GCCATGGACAAGCTGAGGAA |
|  | Reverse | TCGTTATCCCATGTGTCGAAGA |
| EDN1 | Forward | CAGCAGTCTTAGGCGCTGAG |
|  | Reverse | ACTCTT TATCCATCAGGGACGAG |
| IGFBP7 | Forward | ACATCTGGAATGTCACTGGTGC |
|  | Reverse | TACTTCATGCTTTTCTGGGCCA |
| GAPDH | Forward | CTGGTAAAGTGGATATTGTTGCCAT |
|  | Reverse | TGGAATCATATTGGAACATGTAAACC |
| 1#BS (ChIP) | Forward | CTTTCCATCTAGCTGCATCTTTT |
|  | Reverse | TGTCCTGTTGAACAAGTGGTC |
| 2#BS (ChIP) | Forward | GTCTGCATAGCTCCCTGTCC |
|  | Reverse | CAGATTGGGTGGGTAATTGTCC |
| 3#BS (ChIP) | Forward | TCTCAACCCCACGTGTTCTT |
|  | Reverse | TCTCACCTTCCGGATGTTCG |
